# Frequency-dependence in multidimensional diffusion–relaxation correlation MRI of the brain: Overfitting or meaningful parameter?

**DOI:** 10.1162/IMAG.a.143

**Published:** 2025-09-22

**Authors:** Maxime Yon, Omar Narvaez, Jan Martin, Hong Jiang, Diana Bernin, Eva Forssell-Aronsson, Frederik Laun, Alejandra Sierra, Daniel Topgaard

**Affiliations:** Department of Chemistry, Lund University, Lund, Sweden; A.I. Virtanen Institute for Molecular Sciences, University of Eastern Finland, Kuopio, Finland; Department of Chemical Engineering, Chalmers University of Technology, Gothenburg, Sweden; Department of Medical Radiation Sciences, University of Gothenburg, Gothenburg, Sweden; Medical Physics and Biomedical Engineering, Sahlgrenska University Hospital, Gothenburg, Sweden; Department of Radiology, University Hospital Erlangen, Friedrich-Alexander Universität Erlangen-Nürnberg, Erlangen, Germany

**Keywords:** diffusion MRI, frequency-dependent diffusion, multidimensional MRI, tensor-valued diffusion encoding, spectrally modulated gradients, oscillating gradient spin echo (OGSE), Monte Carlo data inversion

## Abstract

Time- or frequency-dependent (“restricted”) diffusion potentially provides useful information about cellular-scale structures in the brain but is challenging to interpret because of intravoxel tissue heterogeneity. Multidimensional diffusion–relaxation correlation MRI with tensor-valued diffusion encoding enables characterization of intravoxel heterogeneity in terms of nonparametric distributions of diffusion tensors and nuclear relaxation rates, and was recently augmented with explicit consideration of frequency-dependence to resolve the effects of restricted diffusion for distinct populations of tissue water. The simplest acquisition protocols for tensor-valued encoding unintentionally cover a frequency range of a factor 2–3, which can be extended in a more controlled way with oscillating gradient waveforms. While microimaging equipment with high-amplitude magnetic field gradients allows exploration of frequencies from tens to hundreds of Hz, clinical scanners with more moderate gradient capabilities are limited to narrower ranges that may be insufficient to observe restricted diffusion for brain tissues. We here investigate the effects of including or omitting frequency-dependence in the data inversion from isotropic and anisotropic liquids, excised tumor tissue, ex vivo mouse brain, and in vivo human brain. For microimaging measurements covering a wide frequency range, from 35 to 320 Hz at *b*-values over 4·10^9^ sm^−2^, the inclusion of frequency-dependence drastically reduces fit residuals and avoids bias in the diffusion metrics for tumor and brain voxels with micrometer-scale structures. Conversely, for the case of in vivo human brain investigated in the narrow frequency range from 5 to 11 Hz at *b* = 3·10^9^ sm^−2^, analyses with and without inclusion of frequency-dependence yield similar fit residuals and diffusion metrics for all voxels. These results indicate that frequency-dependent inversion may be generally applied to diffusion–relaxation correlation MRI data with and without observable effects of restricted diffusion.

## 1 Introduction

MRI provides information about the brain at length scales below the millimeter-scale resolution of the imaging voxels via diffusion metrics (Jones, 2010), reporting on the micrometer-scale organization of macromolecules and cellular membranes acting as barriers for the tissue water (Beaulieu, 2002; Topgaard, 2020), and nuclear relaxation rates (Tofts, 2003) sensitive to the local concentrations of paramagnetic species (Laukien & Schlüter, 1956; Zimmerman, 1954) and chemically exchangeable protons on proteins (Edzes & Samulski, 1975) and carbohydrates (Hills et al., 1989). While quantitative diffusion and relaxation MRI measurements have traditionally been performed separately, recent developments have enabled adaption of multidimensional diffusion–relaxation correlation NMR methods (Bernin & Topgaard, 2013; Galvosas & Callaghan, 2010; Song et al., 2016) to improve characterization of intravoxel heterogeneity in the brain (Benjamini & Basser, 2020; Slator et al., 2021; Tax, 2020). More widespread applications of these new methods in neuroscience studies rely on identification of the most informative acquisition dimensions, design of time-efficient measurement protocols to explore the multidimensional acquisition space, and development of data processing methods with optimal trade-offs between flexibility and risk of overfitting and overinterpretation.

The MRI signal is sensitized to translational motion on the micrometer length scale and the millisecond time scale by application of time-dependent magnetic field gradients. On this length scale, the translational motion in isotropic liquids such as water is fully captured by the self-diffusion coefficient *D* (Morris & Johnson Jr, 1992; Stejskal & Tanner, 1965; Stilbs, 1987), and the relevant acquisition dimension is the “*b*-value” (Le Bihan et al., 1986). For anisotropic materials, the diffusion is described with a tensor **D** (Jost, 1952) which can be determined by performing a series of measurements where the relative orientation between the gradient and the object is varied as demonstrated for clay (Boss & Stejskal, 1965), wood (MacGregor et al., 1983), and brain white matter (WM) (Moseley et al., 1991). Here, the relevant acquisition variable is the encoding tensor **b** (Basser et al., 1994), which can be parameterized in terms of its magnitude, anisotropy, asymmetry, and orientation (Eriksson et al., 2015). In porous rocks (Latour et al., 1993; Woessner, 1963), emulsions (Packer & Rees, 1972; Topgaard et al., 2002), and biological tissues (Cooper et al., 1974; Latour et al., 1994; Tanner, 1979), where the investigated liquid is enclosed in or hindered by micrometer-scale objects, the measurements yield an apparent diffusion coefficient (ADC), which depends on the details of the timing parameters of the motion-encoding gradient waveform—in particular its overall duration which constitutes an additional acquisition variable. The time-dependence of the ADC is often referred to as “restricted” diffusion (Cooper et al., 1974; Packer & Rees, 1972; Stejskal, 1965; Woessner, 1963) and can be further analyzed to extract the surface-to-volume ratio, pore size, and tortuosity of porous media (Latour et al., 1993, 1995). The waveform duration also determines whether the diffusivities of exchanging proton populations can be estimated individually or only as an average (Johnson Jr, 1993; Kärger, 1969).

Conventional diffusion MRI applied to the in vivo human brain is often performed with pairs of magnetic field gradient pulses with durations of tens of milliseconds, corresponding to displacements of a few tens of micrometers. For many tissues, this displacement is much larger than the typical cell sizes and thus yields ADC values that are independent of the experimentally accessible minor variations of the diffusion time and contain aggregated information about local diffusivities, cellular and sub-cellular structures, and barrier properties of the cell membranes (Clark et al., 2001; Le Bihan et al., 1993; Nilsson et al., 2009). Trains of gradient pulse pairs can be used to widen the range over which diffusion is monitored toward shorter time scales and distances (Clark et al., 2001; Schachter et al., 2000; Stepišnik & Callaghan, 2000; Tanner, 1979; Topgaard et al., 2002). While the individual pulse pairs give insufficient diffusion weighting, as quantified by the *b*-value, their effect is accumulated over the duration of the pulse train. The periodicity of such “oscillating gradient spin-echo” (OGSE) diffusion encoding lends itself to analysis with a powerful frequency domain formalism building on tensor-valued diffusion spectra **D**(*ω*) defined as the Fourier transform of the velocity correlation function (Callaghan & Stepišnik, 1995; Stepišnik, 1981). In this case, the tensor-valued encoding spectrum **b**(*ω*) is a useful acquisition variable (Jiang et al., 2023; Lundell & Lasič, 2020; Nielsen et al., 2018; Topgaard, 2019b). In materials with pores having simple and uniform geometries, OGSE can be used to quantify the surface-to-volume ratio (Parsons et al., 2003; Parsons Jr et al., 2006; Reynaud et al., 2016a) and pore sizes (Li et al., 2014; Parsons Jr et al., 2006). Even without extracting quantitative geometrical information, OGSE is useful for providing contrast not available with conventional diffusion methods and has been applied in preclinical MRI at encoding frequencies up to 1 kHz (Portnoy et al., 2013) to highlight specific brain regions, such as the cerebellum or the hippocampus (Aggarwal et al., 2012; Lundell et al., 2015), as well as ischemia (Aggarwal et al., 2014; Does et al., 2003; Wu et al., 2019) and tumors (Colvin et al., 2008, 2011; Reynaud et al., 2016b; Xu et al., 2012). The gradient hardware of clinical MRI systems typically limits the frequency range to 50 Hz which remains sufficient to obtain useful contrast in human brain ( Arbabi et al., 2020; Baron & Beaulieu, 2014; Baron et al., 2015; Tetreault et al., 2020; Van et al., 2014). The accessible frequency range is continuously being extended by further developments of gradient hardware (Dai et al., 2023; Hennel et al., 2021; Michael et al., 2022; Tan et al., 2020).

Interpretation of time- or frequency-dependent ADCs in terms of geometric properties is confounded by heterogeneity of the investigated object on length scales larger than those being mixed by diffusional exchange during the motion-encoding gradients, for instance, multiple tissue types or WM fiber bundles with different orientations within the same imaging voxel. Gradient waveforms with successive diffusion encoding in multiple directions reduce or even remove the effects of diffusion anisotropy on the acquired signal (Mori & van Zijl, 1995) and, when combined with data from conventional unidirectional encoding, enable separation between isotropic and anisotropic sources of intravoxel heterogeneity (Eriksson et al., 2013; Lasič et al., 2014; Szczepankiewicz et al., 2015; Topgaard, 2016b; Westin et al., 2016). By capitalizing on the gain in information content obtained by varying the “shape” (Westin et al., 2014) of the *b*-tensor, the parametric diffusion tensor distribution (DTD) approach (Basser & Pajevic, 2003; Jian et al., 2007; Leow et al., 2009; Magdoom et al., 2021) for describing multi-component diffusion in anisotropic media has been generalized to nonparametric distributions (de Almeida Martins & Topgaard, 2016; Topgaard, 2019a) with in vivo applications in preclinical (Yon et al., 2020) and clinical MRI (Daimiel Naranjo et al., 2021; Reymbaut et al., 2020b). The pros and cons of nonparametric approaches and the corresponding model-based analyses have been extensively discussed in the literature (Benjamini, 2020; Veraart et al., 2020). Combining DTD with diffusion–relaxation correlation (Bernin & Topgaard, 2013; Galvosas & Callaghan, 2010; Song et al., 2016) via variable repetition and/or echo times results in multidimensional correlations between **D** and the relaxation rates *R*_1_ and *R*_2_ (de Almeida Martins & Topgaard, 2018), which has been demonstrated in vivo on small-animal (Rosenberg et al., 2022) and whole-body MRI systems (de Almeida Martins et al., 2020, 2021; Martin et al., 2021; Reymbaut et al., 2021).

Incorporating the sensitivity to restriction of OGSE into the DTD framework (Lundell et al., 2019) yields nonparametric frequency-dependent DTDs or “**D**(*ω*)-distributions” (Narvaez et al., 2024), which allow correlating isotropic and anisotropic diffusivities with restriction in heterogeneous voxels. For mathematical convenience, Narvaez et al. (2024) proposed writing the signal as a sum of contributions from components with axisymmetric diffusion tensors with simple Lorentzian transitions between the low- and high-frequency diffusivities. Although a single Lorentzian produces a sharper frequency transition than those given by the planar, cylindrical, and spherical compartment models (Stepišnik, 1993; Stepišnik et al., 2006), as well as the even smoother power-law frequency-dependence of the random permeable barrier model (Novikov et al., 2011), the multi-Lorentzian approximation was by simulations shown to reproduce the challenging case of the latter model over frequency ranges much larger than practically achievable on any existing MRI system (Narvaez et al., 2024).

While the high-performance gradient hardware of microimaging systems allows comprehensive exploration of both the spectral and tensorial aspects of **b**(*ω*) via “double rotation” gradient waveforms (Jiang et al., 2023), the modest gradient amplitude offered by clinical scanners limits the accessible frequencies to ranges that may not be sufficient to quantify the effects of restriction. Still, tensor-valued encoding on clinical scanners is often associated with a finite frequency range as a side effect of numerical optimization of gradient waveforms to maximize the *b*-value for a range of tensor shapes at constant maximum gradient amplitude and waveform duration without consideration of sensitivity to restriction (Martin et al., 2021; Sjölund et al., 2015). Additionally, most gradient waveforms for “isotropic” or “spherical” diffusion encoding yield a directional-dependence of the spectral content (de Swiet & Mitra, 1996; Lundell & Lasič, 2020). Although typical clinical protocols cover only a factor 2–3 of frequencies, for instance 6–16 Hz in Martin et al. (2021), such a narrow window may in fortuitous cases be sufficient for quantifying the effects of restriction as shown with a 53–160 Hz microimaging protocol for ex vivo rat brain and 44–140 Hz for excised tumor tissue (Narvaez et al., 2024).

OGSE, tensor-valued encoding, and diffusion–relaxation correlation were recently combined into the overarching “massively multidimensional diffusion–relaxation correlation MRI” framework, giving nonparametric **D**(*ω*)-*R*_1_-*R*_2_ distributions (Narvaez et al., 2022). Initial in vivo applications have demonstrated anatomically plausible effects of restriction for rat brain (Yon et al., 2024) but less clear-cut results for human data (Johnson et al., 2024; Manninen et al., 2024). While including the frequency dimension in the data inversion potentially yields useful information, it also comes at the prize of increasing data processing times and the risk of fitting subtle image artifacts rather than signal modulations related to microstructural properties. Conversely, attempting frequency-independent inversion of data that features effects of restriction may result in systematic errors of the estimated parameters (de Swiet & Mitra, 1996; Jespersen et al., 2019).

In this article, we investigate fit residuals and bias of metrics extracted from nonparametric **D**(*ω*)-*R*_1_-*R*_2_ and **D**-*R*_1_-*R*_2_ distributions obtained with (Narvaez et al., 2022) and without (de Almeida Martins & Topgaard, 2018), respectively, inclusion of frequency-dependence in the inversion of experimental data covering wide and narrow frequency ranges. More specifically, we analyze data acquired as a function of echo time, repetition time, and diffusion-encoding gradient waveforms giving *b*-tensor anisotropies (Eriksson et al., 2015) *b*_Δ_ = –0.5 (planar), 0 (spherical), and 1 (linear) using a microimaging system allowing a wide frequency range (35–320 Hz at *b* = 4·10^9^ sm^−2^) for phantoms with well-defined diffusion properties, excised tumor tissue, and fixated ex vivo mouse brain, as well as a conventional clinical system limited to a narrow frequency range (5–11 Hz at *b* = 3·10^9^ sm^−2^) for in vivo human brain. Following previous papers (de Almeida Martins & Topgaard, 2018; de Almeida Martins et al., 2020; Topgaard, 2019a), we tackle the non-uniqueness of the data inversion with a Monte Carlo approach (Prange and Song, 2009) to generate ensembles of distributions from which statistical descriptors can be estimated with acceptable and quantifiable precision (Reymbaut et al., 2020a). Building up for interpretation of the results on frequency-dependence of the water populations in the latter data, we go through the results for the series of simpler systems where mechanisms contributing to frequency dispersion and the mixing of water populations are well known from the chemistry literature, an important message being that the frequency ranges and waveform durations selected mainly because of hardware constraints most likely coincide with some processes in the continuous range of restriction and exchange mechanisms in the living human brain. Since tensor-valued encoding is invariably associated with a finite frequency range, we suggest that frequency-dependence should be included in the data inversion to accommodate the cases where restriction mechanisms happen to fall within the used frequency window. Likewise, interpretation of the obtained results should consider partial or complete mixing of water populations during the waveform durations even when no attempts are made to actually quantify the exchange rates.

## 2 Methods

### 2.1 Theory of multidimensional diffusion and relaxation encoding

**Fig. 1.**
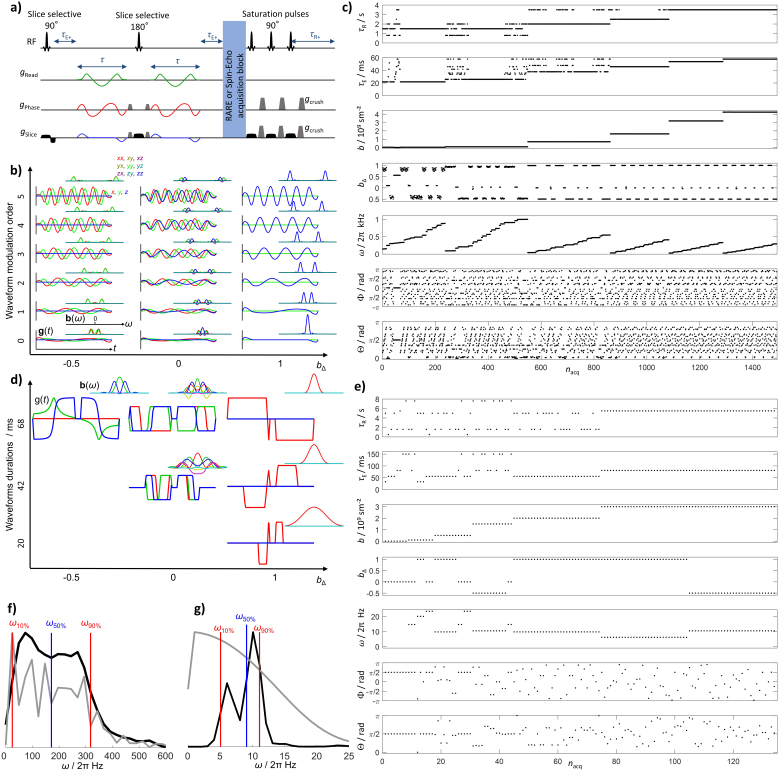
(a) Generic pulse sequence scheme for multidimensional diffusion–relaxation correlation MRI integrating variable echo time *τ*_E_, recovery time *τ*_R_, and time-modulated gradient waveforms **g**(*t*). (b) Double-rotation waveforms and encoding spectra **b**(*ω*), calculated with Eqs. (2)–(4), on the top right used for the preclinical acquisitions for encoding anisotropy *b*_Δ_ = –0.5, 0, and 1, defined in Eq. (10), and up to five oscillations. (c) Preclinical (wide *ω*_cent_-range) acquisition protocol with 1491 images labeled with acquisition number *n*_acq_ and sorted by *b*-value and centroid frequency *ω*_cent_, defined in Eqs. (8) and (9), respectively. (d) Numerically optimized gradient waveforms used for clinical acquisition. (e) Clinical (narrow *ω*_cent_-range) acquisition protocol with 134 image volumes. (f) Total spectral content (gray line) and *b*-weighted *ω*_cent_-distribution (black line) for the preclinical protocol in panel c. Red and blue vertical lines indicate the 10th, 50th, and 90th percentiles of the *ω*_cent_-distribution: *ω*_10%_/2π = 35 Hz, *ω*_50%_/2π = 190 Hz, and *ω*_90%_/2π = 320 Hz. (g) Total spectral content (gray line) and *ω*_cent_-distribution (black line) for the clinical protocol in panel e, yielding *ω*_10%_/2π = 5 Hz, *ω*_50%_/2π = 9 Hz, and *ω*_90%_/2π = 11 Hz. The acquisition protocols in panels c and e are available as tables in the Supplementary Data.

The theory of frequency-dependent and tensor-valued diffusion encoding is described in detail in Lundell and Lasič (2020) and Narvaez et al. (2024), and was incorporated into diffusion–relaxation correlation in Narvaez et al. (2022). As illustrated in Figure 1, data are recorded for numerous combinations of recovery time *τ*_R_ and echo time *τ*_E_, encoding for longitudinal and transverse relaxation, as well as gradient waveforms **g**(*t*) targeting the frequency-dependence and anisotropy of the translational motion. In brief, the signal *S*[**b**(*ω*),*τ*_R_,*τ*_E_] can be expressed as the sum of components *i* characterized by their weights *wi*, tensor-valued diffusion spectra **D***_i_*(*ω*), and relaxation rates *R*_1,_*_i_* and *R*_2,_*_i_* according to

$$S\left[\text{b}\left(\omega \right),\tau_{\text{R}},\tau_{\text{E}}\right]=\sum_{i}w_{i}\text{exp}\left(-\int_{-\infty }^{\infty }\text{b}\left(\omega \right):{\text{D}}_{i}\left(\omega \right)\text{d}\omega \right)\left[1-\exp\left(-\tau_{\text{R}}R_{1,i}\right)\right]\text{exp}\left(-\tau_{\text{E}}R_{2,i}\right),$$ (1)

where the colon indicates a generalized scalar product (Kingsley, 2006) and **b**(*ω*) is the tensor-valued encoding spectrum given by the time-dependent magnetic field gradient **g**(*t*) via the time-dependent dephasing vector **q**(*t*) and its Fourier transform **q**(*ω*) according to

$$\text{q}\left(t\right)=\gamma \int_{0}^{t}\text{g}\left(t^{\prime }\right)\text{d}t^{\prime },$$ (2)

$$\text{q}\left(\omega \right)=\int_{0}^{\tau }\text{q}\left(t\right)\exp\left(\text{i}\omega t\right)\text{d}t,$$ (3)

and

$$\text{b}\left(\omega \right)=\;\frac{1}{2\text{π}}\text{q}\left(\omega \right)\text{q}{\left(-\omega \right)}^{\text{T}}.$$ (4)

In the equations above, *γ* is the gyromagnetic ratio, *τ* is the overall duration of the diffusion-encoding gradients including the imaging gradients, and T denotes a matrix transpose.

In the special case of constant **D***_i_*(*ω*) = **D***_i_* in the investigated frequency interval, Eq. (1) reduces to (de Almeida Martins & Topgaard, 2018)

$$S[\text{b},\tau_{\text{R}},\tau_{\text{E}}]=\sum_{i}w_{i}\text{exp}(-\text{b}:{\text{D}}_{i})[1-\exp(-\tau_{\text{R}}R_{1,i})]\text{exp}(-\tau_{\text{E}}R_{2,i}),$$ (5)

where **b** is the conventional *b*-matrix (Basser et al., 1994) or diffusion-encoding tensor (Westin et al., 2014) given by

$$\text{b}=\overset{\infty }{\underset{-\infty }{\int }}\text{b}\left(\omega \right)\text{d}\omega .$$ (6)

The *ω*-dependent and *ω*-independent tensors **b**(*ω*) and **b** are used in the Monte Carlo inversion of Eqs. (1) and (5) described below. For bookkeeping and to follow common practice in the field, we also convert **b**(*ω*) and **b** to the encoding power spectrum *b*(*ω*) (Callaghan & Stepišnik, 1995), *b*-value (Le Bihan et al., 1986), centroid frequency *ω*_cent_ (Arbabi et al., 2020; Ligneul & Valette, 2017), and normalized anisotropy *b*_Δ_ (Eriksson et al., 2015) via

b(ω)=trace{b(ω)}, (7)

b=trace{b}, (8)

$$\omega_{\text{cent}}=\frac{1}{b}\int_{-\infty }^{\infty }\left|\omega \;\right|b(\omega )\text{d}\omega ,$$ (9)

and

$$b_{\Delta }=\frac{1}{b}\left(b_{ZZ}-\frac{b_{YY}\;+\;b_{XX}}{2}\right),$$ (10)

where *b_XX_*, *b_YY_*, and *b_ZZ_* are the eigenvalues of **b** ordered according to the convention |*b_ZZ_* – *b*/3| > |*b_XX_* – *b*/3| > |*b_YY_* – *b*/3| (Topgaard, 2016b, 2017). The directionality of the encoding is reported as the polar and azimuthal angles, Θ and Φ, of the eigenvector corresponding to the *b_ZZ_* eigenvalue.

For computational convenience, the component tensors are assumed to have axial symmetry:

$${\text{D}}_{i}(\omega )=\text{R}(\theta_{i},\phi_{i})\left(\begin{array}{ccc} D_{\text{R},i}(\omega ) & 0 & 0\\ 0 & D_{\text{R},i}(\omega ) & 0\\ 0 & 0 & D_{\text{A},i}(\omega ) \end{array}\right){\text{R}}^{-1}(\theta_{i},\phi_{i}),$$ (11)

where **R**(*θ*_i_,*ϕ*_i_) is a rotation matrix. Further, the axial and radial eigenvalues *D*_A,_*_i_*(*ω*) and *D*_R,_*_i_*(*ω*) are approximated as Lorentzians (Narvaez et al., 2024),

$$D_{\text{R},i}(\omega )=D_{0,i}-\frac{D_{0,i}-D_{\text{R},i}}{1+\omega^{2}\text{​}/\Gamma_{\text{R},i}^{2}}\;$$ (12)

and

$$D_{\text{A},i}(\omega )=D_{0,i}-\frac{D_{0,i}-D_{\text{A},i}}{1+\omega^{2}\text{​}/\Gamma_{\text{A},i}^{2}},$$ (13)

where *D*_A,_*_i_* and *D*_R,_*_i_* are the low-*ω* diffusivities in the axial and radial directions, respectively, *D*_0,*i*_ is the high-*ω* diffusivity, assumed to be isotropic, and Γ_A,_*_i_* and Γ_R,_*_i_* are the values of *ω* at the mid-points of the transitions. The *ω*-independent case in Eq. (5) corresponds to *D*_A,_*_i_*(*ω*) = *D*_A,_*_i_* and *D*_R,_*_i_*(*ω*) = *D*_R,_*_i_*.

### 2.2 Samples

Saturated salt solutions were prepared by adding Mg(NO_3_)_2_·6 H_2_O and Co(NO_3_)_2_·6 H_2_O (both from Sigma-Aldrich Sweden AB) to H_2_O (Milli-Q quality) until reaching the solubility limits 71 g Mg(NO_3_)_2_ and 97 g Co(NO_3_)_2_ per 100 mL H_2_O (Rumble, 2021). A small amount of Co(NO_3_)_2_ solution (0.27 wt%) was added to the Mg(NO_3_)_2_ solution to increase ^1^H_2_O *R*_1_ and *R*_2_ to approximately 2 and 20 s^–1^, respectively. A lamellar liquid crystal (Ekwall et al., 1969; Jiang et al., 2021) was prepared by mixing 85.79 wt% H_2_O (Milli-Q), 9.17 wt% 1-decanol (Sigma-Aldrich Sweden AB), and 5.04 wt% sodium octanoate (J&K Scientific via Th. Geyer in Sweden). The composite phantom was assembled by inserting 4-mm NMR tubes containing salt solution and liquid crystal into a 10-mm NMR tube with H_2_O.

The excised tumor tissue was obtained by culture of human neuroblastoma cells grown at 37°C and 5% CO_2_ in a complete medium (RPMI 1640 supplemented with 10% fetal bovine serum and 1% penicillin/streptomycin). Approximately 2·10^6^ of those tumor cells were subcutaneously inoculated to a female BALB/c mouse (Janvier Labs, France). The mouse was sacrificed after 5 weeks of tumor growth and the tumor was removed and immediately transferred to a 10-mm NMR tube containing 4% paraformaldehyde (PFA) in phosphate buffer solution (Histolab, Sweden). The sample was stored at room temperature for several years before being investigated with MRI.

The ex vivo mouse brain was obtained from an 8-week-old C57BL/6 female mouse intracardially perfused with 0.9% saline followed by 4% PFA fixation. The brain was carefully extracted from the skull and stored in 2% PFA solution at 4ºC before MRI acquisition. The procedure was approved by the Animal Committee of the Provincial Government of Southern Finland following the guidelines established by the European Union Directives 2010/63/EU.

The human data were obtained with informed consent on a healthy young adult with approval from the local institutional review board.

### 2.3 MRI acquisition and reconstruction

The composite phantom, excised tumor tissue, and ex vivo mouse brain were investigated using a Bruker Avance Neo spectrometer (Bruker Biospin, Karlsruhe, Germany) with an 11.7 T magnet, an MIC-5 probe delivering 3 Tm^–1^ maximum gradient strength, and a 10 mm ^1^H radiofrequency coil. Images were acquired in Paravision 360 v1.1 with custom-made sequences based on either Rapid Acquisition with Relaxation Enhancement (RARE) (Hennig et al., 1986) or multi-slice multi-echo (MSME) (Edelstein et al., 1980) with spin-echo diffusion preparation according to the generic pulse sequence scheme presented in Figure 1a. The mouse brain MSME images were acquired at 25°C sample temperature with 14 × 9 × 0.5 mm^3^ field of view (FOV) and 100 × 64 × 1 reconstructed image size, giving 140 × 140 × 500 µm^3^ resolution. Using a single scan per each of the 36 phase-encoding steps with 1.8 partial Fourier factor led to an acquisition time of 36 h and 8 min. The phantoms and tumor RARE images were acquired at 20°C sample temperature with 12 × 12 × 0.5 mm^3^ FOV and 64 × 64 × 1 matrix size, giving 190 × 190 × 500 µm^3^ resolution. A partial Fourier factor of 1.8 was used in the first phase dimension to reduce the echo time and allow single-shot acquisition with 36 echoes. Using four averages led to an acquisition time of 4 h and 7 min. Anisotropic voxel sizes were used to reach acceptable signal-to-noise ratio (SNR) at high in-plane image resolution. The images were reconstructed with Paravision 360 v1.1, followed by denoising (Cordero-Grande et al., 2019) implemented in MRTrix3 (Tournier et al., 2019), as well as Gibbs ringing removal (Kellner et al., 2016) for the mouse brain dataset.

The in vivo data were recorded using a Siemens Magnetom Prisma (Siemens Healthineers AG, Erlangen, Germany) with a 3 T magnet, a gradient system providing a maximum amplitude of 0.08 Tm^–1^, and a 20-channel head coil. The images were acquired with a single-shot spin echo-echo planar imaging (SE-EPI) sequence customized for general gradient waveforms (Martin et al., 2020; Wetscherek et al., 2015). The acquisition parameters were 230 × 230 mm² FOV, 3 mm^3^ isotropic resolution, 30 slices in axial orientation, 1496 Hz/Px readout bandwidth, and a factor 3 acceleration with GRAPPA reconstruction. The total measurement time was 20 min. The data were preprocessed with denoising (Cordero-Grande et al., 2019; Tournier et al., 2019), removal of Rician noise baseline (Koay & Basser, 2006) and Gibbs ringing (Kellner et al., 2016), and motion and eddy current correction (Klein et al., 2010; Nilsson et al., 2015).

### 2.4 Multidimensional diffusion and relaxation acquisition protocols

In the preclinical system, diffusion encoding was performed with two identical self-refocusing gradient waveforms, while in the clinical setting, the waveforms were different and not self-refocused to maximize the *b*-value per encoding time unit (Sjölund et al., 2015). Gradient waveforms and the corresponding encoding spectra **b**(*ω*) at *b*_Δ_ = –0.5, 0, and 1 are shown in Figure 1b and d for the preclinical and clinical scanners, respectively. In the preclinical setting, the gradient waveforms were generated by double rotation of the *q*-vector (Jiang et al., 2023) and the *ω*_cent_ dimension was explored over a wide range by varying the number of oscillations from 0 to 5 and the waveform duration from 4 to 22 ms as shown in Figure 1b. The values of *b* and *ω*_cent_ for each acquired image volume are shown in Figure 1c, illustrating that the highest values of *ω*_cent_ are achieved for low *b*-values only. The frequency-modulated gradient waveforms were normalized to give constant *b*-values at identical waveform lengths, allowing us to map the entire *b*-value space (from 0.033 to 4.25·10^9^ sm^−2^) for all number of oscillations. The deviations of *b*_Δ_ from the target values –0.5, 0, and 1 at low *b*-values result from the imaging gradients which were all taken into account when computing **b**(*ω*) via Eqs. (2)–(4) above. In the clinical setting, the limited maximum gradient amplitude necessitated numerical optimization of the waveforms (Sjölund et al., 2015) to reach *b* = 3·10^9^ sm^−2^ at *τ*_E_ = 83 ms and *b*_Δ_ = –0.5, 0, and 1 as previously used in Martin et al. (2020) and Martin et al. (2021). The directional-dependence of the diffusion was probed by rotating the waveforms in Figure 1b and d according to the angles Θ and Φ in Figure 1c and e.

Figure 1f and g shows quantitative assessments of the range of frequencies investigated in each acquisition protocol in terms of the *b*-weighted *ω*_cent_-distribution (black lines) and the total spectral content (gray lines) expressed as the sum of *b*(*ω*) over all acquisitions with index *n*_acq_. For the preclinical protocol (Fig. 1f), each waveform yields *b*(*ω*) focused on a narrow frequency range centered on *ω*_cent_, and the width of the total spectral content matches the one of the broad *ω*_cent_-distribution, implying that the protocol is appropriate for investigating frequency-dependence. Conversely, each clinical waveform yields a poorly defined *b*(*ω*) with width that surpasses the spread of *ω*_cent_ across the acquisitions, resulting in total spectral content far broader than the narrow *ω*_cent_-distribution (Fig. 1g) and data that are less amenable for extracting frequency-dependence.

The preclinical protocol of 1491 images includes variable *τ*_R_ between 0.8 and 3.5 s as well as variable *τ*_E_ between 13 and 49 ms for MSME and 22 to 58 ms for RARE, the latter being displayed in Figure 1c. The clinical protocol with 134 image volumes includes *τ*_R_ from 0.5 to 7.6 s and *τ*_E_ from 33 to 150 ms. Values of *τ*_R_ below 1.6 s were reached by acquiring the 30 slices in packages with a few slices each. The minimum value of *τ*_E_ is constrained by the durations of the imaging and diffusion-encoding gradients. These durations are determined by the gradient hardware capabilities and the choice of spatial resolution, *b*-value, and *ω*_cent_ (Jiang et al., 2023). The maximum *τ*_E_ and minimum *τ*_R_ are selected to reach nearly complete signal attenuation to the noise baseline. The maximum *τ*_R_ is chosen to reach almost full signal recovery by longitudinal relaxation.

### 2.5 Monte Carlo data inversion

A Monte Carlo algorithm implemented in the *md-dmri* (Nilsson et al., 2018) Matlab toolbox was used to estimate ensembles of discrete distributions in the spaces [*D*_A_,*D*_R_,*θ*,*ϕ*,*D*_0_,Γ_A_,Γ_R_,*R*_1_,*R*_2_] (Narvaez et al., 2022) and [*D*_A_,*D*_R_,*θ*,*ϕ*,*R*_1_,*R*_2_] (de Almeida Martins & Topgaard, 2018), corresponding to Eqs. (1) and (5), respectively. Using the terminology in Reymbaut et al. (2020a), the inversion was performed with 20 steps of proliferation, 20 steps of mutation/extinction, 200 input components per step of proliferation and mutation/extinction, and 10 output components. Bootstrapping was performed by 100 repetitions using random sampling with replacement. Preclinical (clinical) data were inverted with the parameter limits 5·10^−12^ m^2^s^−1^ < *D*_0/A/R_ < 5·10^−9^ m^2^s^−1^, 0.1 s^−1^ < Γ_A/R_ < 10^5^ s^−1^, 0.1 s^−1^ < *R*_1_ < 4 s^−1^, and 4 s^−1^ < *R*_2_ < 150 s^−1^ (5·10^−11^ m^2^s^−1^ < *D*_0/A/R_ < 5·10^−9^ m^2^s^−1^, 0.1 s^−1^ < Γ_A/R_ < 10^4^ s^−1^, 0.2 s^−1^ < *R*_1_ < 2 s^−1^, and 1 s^−1^ < *R*_2_ < 30 s^−1^). The influence of noise on distributions obtained for synthesized data can be found in the Appendix.

### 2.6 Quantitative parameter distributions and maps

To enable visualization, the distributions in the primary analysis space [*D*_A_,*D*_R_,*θ*,*ϕ*,*D*_0_,Γ_A_,Γ_R_,*R*_1_,*R*_2_] were evaluated at selected values of *ω*, using Eqs. (12) and (13), and projected onto the dimensions of isotropic diffusivity *D*_iso_(*ω*) and squared normalized diffusion anisotropy *D*_Δ_^2^(*ω*) (Conturo et al., 1996; Eriksson et al., 2015; Topgaard, 2019a) via

$$D_{\text{iso},i}(\omega )=\frac{D_{\text{A},i}(\omega )+2D_{\text{R},i}(\omega )}{3}$$ (14)

and

$$D_{\Delta ,i}^{2}(\omega )={\left(\frac{D_{\text{A},i}(\omega )-D_{\text{R},i}(\omega )}{D_{\text{A},i}(\omega )+2D_{\text{R},i}(\omega )}\right)}^{2},$$ (15)

respectively, yielding *ω*-dependent distributions in the [*D*_iso_(*ω*),*D*_Δ_^2^(*ω*),*θ*,*ϕ*,*R*_1_,*R*_2_] space. The use of *D*_Δ_^2^ rather than *D*_Δ_ reduces the risk of microstructural overinterpretation at values of |*D*_Δ_| below 0.5 were both oblate *D*_Δ_ < 0 and prolate *D*_Δ_ > 0 tensor shapes may be consistent with acquired data (Eriksson et al., 2015). Projections onto the 2D *D*_iso_-*D*_Δ_^2^ plane (Topgaard, 2019a) were obtained by mapping the weights *w_i_* of the discrete components onto a 64 × 64 mesh using a 3 × 3 grid points Gaussian kernel. Image segmentation was performed by dividing the 2D *D*_iso_-*D*_Δ_² space into three bins with diffusion properties characteristic for white matter (WM, bin1), gray matter (GM, bin2), and cerebrospinal fluid (CSF, bin 3) using the limits bin1: *D*_iso_ < 1·10^−9^ m^2^s^−1^ and *D*_Δ_^2^ > 0.25; bin2: *D*_iso_ < 1·10^−9^ m^2^s^−1^ and *D*_Δ_^2^ < 0.25; and bin3: *D*_iso_ > 1·10^−9^ m^2^s^−1^ (bin1: *D*_iso_ < 2·10^−9^ m^2^s^−1^ and *D*_Δ_^2^ > 0.25; bin2: *D*_iso_ < 2·10^−9^ m^2^s^−1^ and *D*_Δ_^2^ < 0.25; and bin3: *D*_iso_ > 2·10^−9^ m^2^s^−1^) for the ex vivo mouse (in vivo human) data and calculating bin-resolved signal fractions *f*_bin_*_n_* by

$$f_{\text{bin}n}=\frac{1}{S_{0}}\sum_{i\in \text{bin}n}w_{i},$$ (16)

where

$$S_{0}=\sum_{i}w_{i}$$ (17)

is the signal extrapolated to *b* = 0, *τ*_R_ = ∞, and *τ*_E_ = 0. The values of *f*_bin_*_n_* were converted to RGB color via

$$\left[\text{R},\text{G},\text{B}\right]=\frac{\left[f_{\text{bin}1},f_{\text{bin}2},f_{\text{bin}3}\right]}{\text{max}(f_{\text{bin}1},f_{\text{bin}2},f_{\text{bin}3}\text{)}}.$$ (18)

The rich information in the [*D*_iso_(*ω*),*D*_Δ_^2^(*ω*),*θ*,*ϕ*,*R*_1_,*R*_2_]-distributions was further condensed into means E[*X*] over selected dimensions according to Topgaard (2019a)

$$\text{E}\left[X\right]=\frac{1}{S_{0}}\sum_{i}w_{i}X_{i},$$ (19)

where *X* symbolizes *D*_iso_(*ω*) and *D*_Δ_^2^(*ω*) at the frequencies *ω*_10%_, *ω*_50%_, and *ω*_90%_ indicated in Figure 1f and g, as well as *R*_1_ and *R*_2_. The dispersion of the diffusion metrics within the investigated frequency window Δ*_ω_*_/2π_E[*X*] was calculated through (Aggarwal et al., 2012; Narvaez et al., 2024)

$$\Delta_{\omega /2\text{π}}\text{E}\left[X\right]=\frac{\text{E}\left[X(\omega_{90\text{\%}})\right]-\text{E}\left[X(\omega_{10\text{\%}})\right]}{(\omega_{90\text{\%}}-\omega_{10\text{\%}})/2\text{π}}.$$ (20)

To evaluate the uncertainty of the data inversion procedure, the diffusion- and relaxation-encoded signals *S*[**b**(*ω*),*τ*_R_,*τ*_E_], 2D *D*_iso_-*D*_Δ_^2^ projections, extrapolated signals *S*_0_, signal fractions *f*_bin_*_n_*, means E[*X*], and dispersions Δ*_ω_*_/2π_E[*X*] were calculated independently for each of the 100 bootstrap replicates. The values underlying the graphs and maps in the following figures were obtained as medians over these 100 replicates. Rough estimates of the per-voxel SNR were obtained by taking the ratio between *S*_0_ and the standard deviation of the difference between the measured and back-calculated *S*[**b**(*ω*),*τ*_R_,*τ*_E_].

For the *ω*-independent analysis based on Eq. (5), the metrics in Eqs. (14)–(19), including the binning, was performed with the *ω*-independent values of *D*_A_ and *D*_R_ obtained directly in the primary analysis space [*D*_A_,*D*_R_,*θ*,*ϕ*,*R*_1_,*R*_2_]. Comparison between the *ω*-dependent and *ω*-independent results was performed by evaluating the former at the center of the investigated frequency window, corresponding to the value *ω*_50%_ labeled in Figure 1f and g, and computing the normalized difference via

$$\text{normalized difference}=\frac{Y_{\omega -\text{dependent}}-Y_{\omega -\text{independent}}}{\left(Y_{\omega -\text{dependent}}+Y_{\omega -\text{independent}}\right)\;/\;2}\times 100\%,$$ (21)

where *Y* represents *S*_0_, E[*D*_iso_], E[*D*_Δ_^2^], E[*R*_1_], E[*R*_2_], or *f*_bin_*_n_*.

### 2.7 Regions of interest

Regions of interest (ROIs) were manually delineated to exhibit homogeneous and pure signatures of the various chemicals and tissues. We chose their locations and sizes to ensure that all the selected voxels within each ROI contained an equivalent signature. Within such conditions, increasing the ROI size increases the effective SNR. The parameter distributions of the ROIs were obtained by projecting all the voxel weights *w_i_* of the discrete components onto the 2D *D*_iso_-*D*_Δ_^2^ plane using a 3 × 3 grid points Gaussian kernel.

## 3 Results and Discussion

**Fig. 2.**
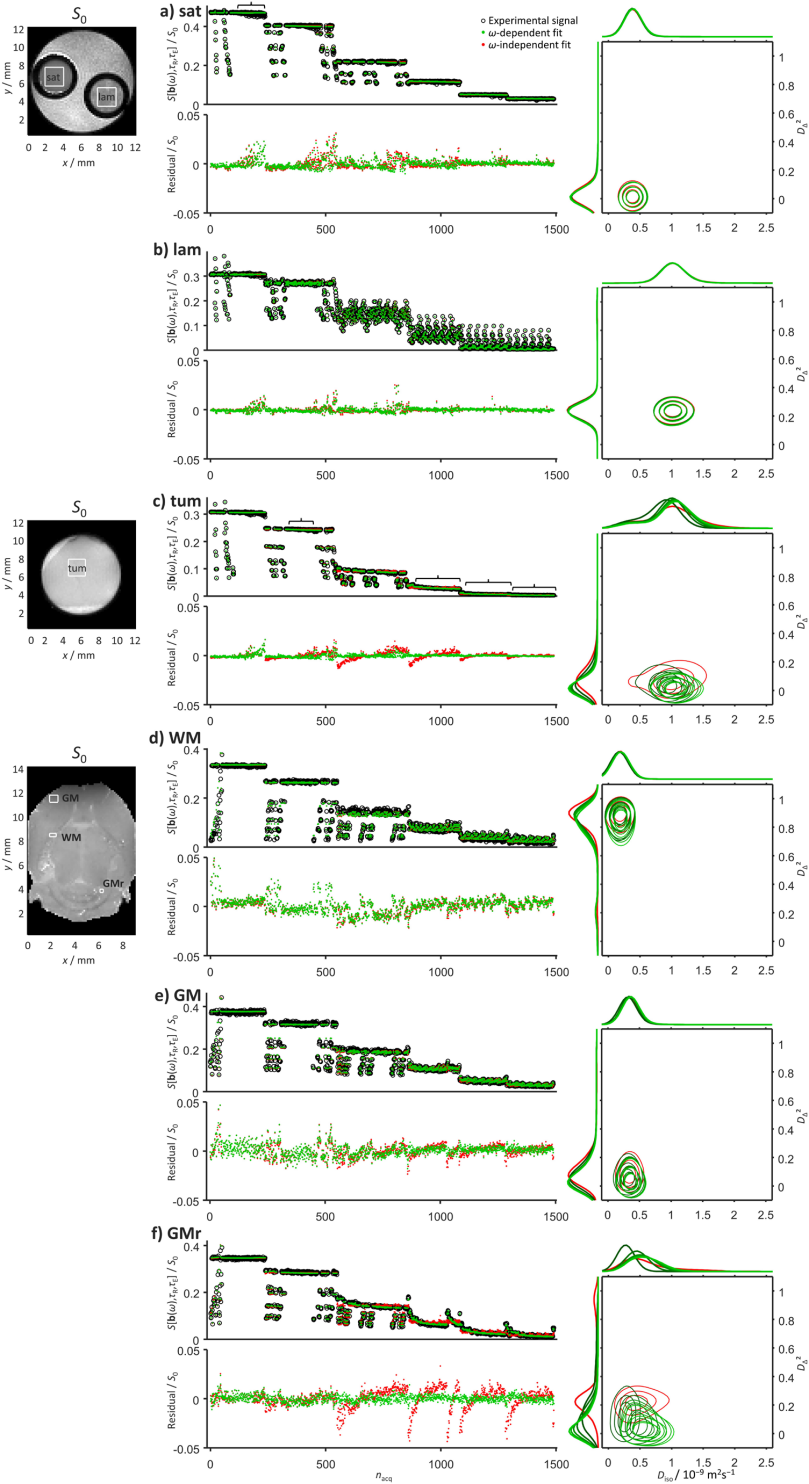
Comparison between *ω*-dependent and *ω*-independent data inversion results for illustrative cases with distinct water diffusion properties. (a) Isotropic Gaussian diffusion in an aqueous solution saturated with magnesium nitrate salt (sat). (b) Planar anisotropic Gaussian diffusion in a lamellar liquid crystal (lam). (c) Isotropic restricted diffusion in tumor tissue (tum). (d) Linear anisotropic Gaussian diffusion in white matter (WM) of the internal capsule. (e) Isotropic restricted diffusion in the gray matter (GM) of the cortex. (f) Isotropic restricted diffusion in the gray matter of the cerebellum (GMr). Panels to the left show labeled regions of interest (ROIs) for the composite phantom (lam and sat), excised tumor (tum), and ex vivo mouse brain (WM, GM, and GMr) on maps of the signal *S*_0_ extrapolated to *b* = 0, *τ*_R_ = ∞, and *τ*_E_ = 0, see Eq. (17). The center panels display measured signals *S*[**b**(*ω*),*τ*_R_,*τ*_E_] versus acquisition number *n*_acq_ according to the preclinical (wide *ω*_cent_-range) protocol in Figure 1c (black circles), signals back-calculated from the distributions obtained by Monte Carlo inversion of the *ω*-dependent (green dots) and *ω*-independent (red dots) expressions in Eqs. (1) and (5), respectively, as well as residuals given by the differences between the measured and back-calculated signals. Signals and residuals are normalized with *S*_0_. The right part of the figure presents the *ω*-dependent (green) and *ω*-independent (red) distributions as projections onto the 2D *D*_iso_-*D*_Δ_^2^ plane (contour plots) as well as the 1D *D*_iso_ and *D*_Δ_^2^ dimensions (horizontal and vertical line plots sharing axes with the 2D plots). The *ω*-dependence is illustrated by overlaying color-coded plots for 5 linearly spaced values of *ω*/2π between 35 (dark green) and 320 Hz (pale green). Contours extending slightly below *D*_Δ_^2^ = 0 originate from the 3 × 3 Gaussian kernel used to map from ensembles of discrete components to the 64 × 64 mesh in the 2D *D*_iso_-*D*_Δ_^2^ projection. Overbraces in panels a and c point out protocol sections at constant *b*,*τ*_R_, and *τ*_E_ where the residuals are unaffected or decrease, respectively, by including *ω*-dependence in the inversion.

Figure 2 shows data for a series of ROIs in samples with distinct restriction and anisotropy characteristics investigated using the preclinical protocol shown in Figure 1c. The selected samples represent chemically “simple” systems, where the relevant time scales and mechanisms affecting the observed diffusivities are well understood from the chemistry literature, as well as a series of biomedically more interesting tissue ROIs where the contributing mechanisms are the same as for the previous examples, but the relevant time scales are more difficult to predict due to the increased chemical complexity and the continuous range of structural organization levels from the molecular to the macroscopic.

According to the literature, the saturated magnesium nitrate solution in panel a exhibits a water diffusivity of 0.44·10^−9^ m^2^s^−1^ at 25ºC (Wadsö et al., 2009), which is 20% of the value 2.3·10^−9^ m^2^s^−1^ for pure water (Mills, 1973) on account of interactions between the water and the ions. The chemical composition reported in the Methods section can be converted to a molar ratio of 12 water molecules per magnesium ion and 2 nitrate ions, implying that every water molecule is in direct atomic-level contact with neighboring ions. The water–ion electrostatic interactions are dominated by the magnesium ion because of its higher charge density, two positive charges for an ionic radius of 86 ppm, compared with the nitrate ion with one negative charge for a thermochemical radius of 179 ppm (Simoes et al., 2017). In dilute solution, the first hydration shell of the magnesium ion comprises six water molecules with a lifetime of about 1 μs before exchange with the less well-defined and more labile second hydration shell and surrounding bulk water (Bleuzen et al., 1997; Neely & Connick, 1970). Including the <10^–12^ s decay of the velocity autocorrelation function for pure water (Balucani et al., 1996) and the >10^3^ s time required for the mean squared displacement to equal the distance between the walls of the 4 mm glass tube, we would thus expect the diffusion spectrum **D**(*ω*) to show *ω*-dependence at the widely space values 10^–3^, 10^6^, and 10^12^ Hz, but not within the ~30–300 Hz range defined by the currently used gradient waveforms with ~50 ms total duration. Returning to Figure 2a, the expectations of isotropic Gaussian diffusion are borne out by the absence of signal modulations from the acquisition variables *ω*_cent_, *b*_Δ_, Θ, and Φ at constant *b*, *τ*_R_, and *τ*_E_, as well as nearly identical fit residuals for data inversions based on the *ω*-dependent and *ω*-independent expressions Eqs. (1) and (5), respectively. The corresponding 2D *D*_iso_-*D*_Δ_² projections of the obtained distributions comprise a single peak at *D*_iso_ = 0.4·10^−9^ m^2^s^−1^ and *D*_Δ_² = 0 with no detectable *ω*-dependence. The peak width originates mainly from the variability of the 100 replicate solutions obtained by the bootstrapping and Monte Carlo data inversion (Reymbaut et al., 2020a), which includes neither the conventional Tikhonov regularization (Provencher, 1982; Whittal & MacKay, 1989), leading to peak broadening, nor sparsity constraints (Aranda et al., 2015; Berman et al., 2013) favoring narrow peaks. Our results at 20ºC are consistent with literature data at 25ºC (Wadsö et al., 2009) and should be interpreted as an average over exchanging water populations in the first and second hydration shells of the magnesium ions with no influence from restriction by the glass tube walls. More detailed comparison between the plots of residuals in Figure 2 and the protocol in Figure 1c shows that acquisitions above 500 Hz yield data that cannot be fully captured even by the *ω*-dependent expression in Eq. (1) despite the *b*-value being too low to give any appreciable diffusion weighting, indicating that these data points are corrupted by image artifacts with magnitude of a few percent that may be difficult to detect by visual inspection of the raw images. Although these data points (overbrace in panel a) should be excluded in improved versions of the protocol, they appear to have negligible influence on the obtained distributions and are useful in the context of this work as a reference for whether or not inclusion of *ω*-dependence in the inversion improves the analysis.

The lamellar liquid crystal in Figure 2b consists of stacks of decanol and sodium octanoate bilayers separated by water. The bilayers have a thickness of 2.5 nm (Ekwall et al., 1969), a lamellar repeat distance of 15 nm (Jiang et al., 2021), and are organized with the hydrophilic hydroxyl and carboxylate groups facing the water and hydrophobic hydrocarbon chains in the bilayer interior. The sodium ions are distributed across the 12.5 nm thick water layers with preferential location within a few nm distance from the bilayer surface on account of electrostatic interactions with the oppositely charged carboxylate groups (Evans & Wennerström, 1999). There are six water molecules within the first hydration shells of both divalent magnesium and monovalent sodium ions, but the lifetime is only ~1 ns (Helm & Merbach, 1999) for the latter species because of the lower charge density (one positive charge for an ionic radius of 116 pm). Additionally, proton exchange between decanol hydroxyl groups and water takes place on time scales lower than 1 ms (Hills, 1990). The chemical composition of the liquid crystal corresponds to ~160 water and 2 decanol molecules per sodium and octanoate ion pair, and, as opposed to the case of the saturated salt solution, only a small fraction of the water molecules is in direct atomic-scale contact with the ions or bilayers. The low water concentration within the hydrophobic interior of the bilayers makes them efficient barriers for water diffusion (Evans & Wennerström, 1999), and the time for the mean squared displacement to cover the gap between two adjacent bilayers is ~40 ns. The lateral extension of the bilayers may approach macroscopic length scales and is ultimately limited by the walls of the glass tube (Bernin et al., 2014; Topgaard, 2016a), leading to characteristic time scales above 10^2^ s for diffusional exchange between differently oriented bilayer sections having a radius of curvature above 1 mm (Lutti & Callaghan, 2007). Partial alignment of the chain-like decanol molecules and octanoate ions in the direction of the bilayer normal vector renders the motional averaging of intermolecular ^1^H-^1^H dipolar couplings incomplete (Wennerström, 1973), leading to transverse relaxation on time scales shorter than the ~20 ms minimum echo time in the current protocol and minimal contribution from these species to the intensity of the detected images. Taken together, we may thus anticipate *ω*-dependence of **D**(*ω*) at multiple frequencies including 10^–2^ (water diffusion along bilayer curvature), 10^3^ (water–decanol chemical exchange), 10^8^ (diffusion across water layers), 10^9^ (lifetime of water in sodium ion hydration layer), and 10^12^ Hz (transition from ballistic to diffusive regime of pure water), none of which being located within the narrow *ω*-range explored with the present gradient waveforms. Expectedly, the data for the liquid crystal in Figure 2b show pronounced signal modulations as a function of the *b*_Δ_, Θ, and Φ acquisition variables, which by itself indicates anisotropy, but no differences in fit residuals between the *ω*-dependent and *ω*-independent inversions, showing that the diffusion is Gaussian in the investigated window. The highest fit residuals of a few percent are found at low-*b* and high-*ω*_cent_ acquisitions and presumably originate from the minor image artifacts previously discussed for the salt solution. The 2D *D*_iso_-*D*_Δ_² projections feature a single *ω*-independent peak at *D*_iso_ = 1.0·10^−9^ m^2^s^−1^ and *D*_Δ_² = 0.25, corresponding to *D*_A_ << *D*_R_ and *D*_R_ = 1.5·10^−9^ m^2^s^−1^. The latter value describes lateral diffusion along the planes of the bilayers and is given by a ~50 ms time average over protons in multiple exchanging populations including pure water in the center of the water layers, water in the hydration shells of the sodium ions and hydrophilic groups at the surfaces of the bilayers, and the hydroxyl groups of the decanol molecules. Additionally, the diffusion of the water molecules may be hindered by the roughness of the hydrophobic–hydrophilic interface originating from molecular protrusions (Ben-Shaul & Gelbart, 1985) or bilayer undulations (Lindahl & Edholm, 2000), leading to an observed lateral diffusivity that is 75% of the value 2.0·10^−9^ m^2^s^−1^ for pure water at 20ºC (Holz et al., 2000).

The data in Figure 2c are obtained on excised tumor tissue preserved in paraformaldehyde solution. Despite the increase in chemical and structural complexity, the basic mechanisms contributing to lowering the observed diffusivity compared with the reference state of pure water are the same as for the simpler systems in panels a and b, namely proton exchange between water and labile functional groups, hydration of ions, and obstruction by larger molecules and aggregates. While the importance of the two first mechanisms could in principle be estimated by detailed analysis of the chemical composition of the tissue sample (Persson & Halle, 2008), the experimental observations are dominated by the latter mechanism which depends critically on the details of how the molecules are spatially arranged—in particular the assembly of lipids into bilayers that may or may not be efficient barriers for water diffusion (Topgaard, 2020). From the perspective of water dynamics, biological tissues can conceptually be divided into numerous subvolumes with different local concentrations of everything from small ions and metabolites to large macromolecules and macromolecular assemblies that influence water motion via the mechanisms of hydration and obstruction. Some of these subvolumes are formed by the thermodynamic equilibrium mechanism of liquid–liquid phase separation into regions with low and high concentration of macromolecules (Banani et al., 2017; Hyman et al., 2014). Other subvolumes are formed by semipermeable biomembranes that encapsulate regions of space with different chemical compositions than the surroundings and prevent equilibration of concentration gradients. The subvolumes—with or without biomembrane enclosure—have dimensions evenly spread out within the 10 nm to 100 μm range that is of relevance for rationalizing diffusion data, some examples being vesicles, condensate droplets, lysosomes, mitochondria (with internal compartmentation), endoplasmic reticulum, nucleus, cytosol, and the extracellular space. Because of the varying barrier properties of the biomembranes and the multiple structural levels in the tumor tissue, we may expect diffusional exchange of water between the subvolumes on a continuous range of time scales as well as a continuous *ω*-dependence of **D**(*ω*) filling in the gaps of the numerous processes from 10^–2^ to 10^12^ Hz described above for the salt solution and lamellar liquid crystal. In some aspects, the signal data for the tumor in panel c resemble the one from the salt solution in panel a, showing minor influence of the acquisition variables Θ and Φ at constant *b*, *τ*_R_, and *τ*_E_, consistent with isotropic diffusion, as well as elevated residuals originating from image artifacts in the low *b* and excessively high *ω*_cent_ range of the data. At higher *b*, the tumor data display a marked dependence of the signal on *ω*_cent_ and greatly improved fit residuals with the *ω*-dependent analysis (overbrace in panel c) indicating *ω*-dependence of **D**(*ω*) within the investigated window ~30–300 Hz. The corresponding *ω*-dependent 2D *D*_iso_-*D*_Δ_² projections show a single peak moving from E[*D*_iso_] = 0.84·10^−9^ m^2^s^−1^ and E[*D*_Δ_²] = 0.050 at 35 Hz to E[*D*_iso_] = 1.1·10^−9^ m^2^s^−1^ and E[*D*_Δ_²] = 0.014 at 320 Hz. Although the observed values for E[*D*_iso_] can be reproduced by inserting 1.2·10^−9^ m^2^s^−1^ local diffusivity and 7 μm radius in the model for restricted diffusion in a closed spherical compartment (Stepišnik, 1993), we emphasize that such a geometric interpretation is most certainly an oversimplification that may give rise to misconceptions if applied by users not familiar with the underlying assumptions and the plethora of alternative models with equal ability to describe the experimental results. Without overinterpretation, we may state that all detectable proton populations with potentially distinct diffusion properties are mixed on the ~50 ms time scale of the diffusion-encoding gradients, giving rise to a single peak in the 2D *D*_iso_-*D*_Δ_² projections, and that these exchange-averaged populations experience nearly isotropic structural barriers on the length scale of a few micrometers. Converting these imprecise statements to quantitative information about, for example, compartment sizes and shapes, barrier permeabilities, and local diffusivities, would require model assumptions that are difficult to justify in light of the known chemical and structural complexity.

The remaining panels d, e, and f in Figure 2 present results for three regions of the ex vivo mouse brain: white matter (WM) in the internal capsule, gray matter in the cortex (GM), and gray matter in the cerebellum (GMr). WM consists of closely packed aligned axons with ~0.1–10 μm diameters (Saliani et al., 2017) and lengths on the macroscopic scale. The axons are piecewise enclosed by myelin sheaths formed by multilayer wraps of oligodendrocyte cell membranes. In addition to the numerous cellular-scale subvolumes, WM can on a coarser level be divided into the intraaxonal and extracellular spaces, as well as the myelin sheaths which in themselves have intra- and extracellular spaces. All these subvolumes have distinct types of organization of macromolecular assemblies and biomembranes that determine the local diffusion properties of the water. In analogy with the reasoning for the tumor tissue, we may expect some exchange or restricted diffusion process to occur at any given time scale. The data for WM in panel d share some distinguishing features with the liquid crystal in Figure 2b: signal fluctuations with acquisition variables *b*_Δ_, Θ, and Φ at constant *b*, *τ*_R_, and *τ*_E_, but only minor differences in fit residuals between the *ω*-dependent and *ω*-independent inversions. These observations indicate anisotropic diffusion without detectable *ω*-dependence within the investigated ~30–300 Hz window. The corresponding 2D *D*_iso_-*D*_Δ_² projection comprises a single peak at E[*D*_iso_] = 0.2·10^−9^ m^2^s^−1^ and E[*D*_Δ_²] moving slightly from 0.89 at 35 Hz to 0.85 at 320 Hz. The presence of just one peak shows that exchange averaging over the ~50 ms duration of the gradient waveform has rendered the detectable water populations too similar to resolve within the variability of the 2D *D*_iso_-*D*_Δ_² projections of the 100 replicate solutions obtained by bootstrapping and Monte Carlo data inversion. According to Eq. (15), the value of *D*_Δ_² reaches unity in the extreme case of *D*_A_ >> *D*_R_ and *D*_R_ = 0, corresponding to one-dimensional diffusion in an infinitesimally thin cylinder. The observed values of E[*D*_Δ_²] are close to one-dimensional diffusion, but still accommodate sufficient displacements in the radial directions to mix water populations in the intraaxonal and adjacent extracellular spaces via the gaps between the myelin patches or directly across the sheaths (Le Bihan et al., 1993). Alternatively, the observations are consistent also with a scenario where the populations remain separate but coincidentally have too similar values of both *D*_iso_ and *D*_Δ_² to resolve without postulating their existence as in the popular model-based approaches (Assaf & Basser, 2005; Zhang et al., 2012).

GM comprises mainly neuronal cell bodies, glial cells, and nonmyelinated axons with low orientational order. The cortex and cerebellum GM data in Figure 2e and f resemble the tumor data in Figure 2c, with negligible influence of Θ and Φ at constant *b*, *τ*_R_, and *τ*_E_, indicating isotropic diffusion. The deviations between the residuals from the *ω*-dependent and *ω*-independent analyses are clearly visible for both the cortex and cerebellum, but the magnitude is larger for the latter case indicating more pronounced *ω*-dependence in the ~30–300 Hz range. Both examples feature single peaks in the 2D *D*_iso_-*D*_Δ_² projections, with a shift of the peak maximum with frequency being readily apparent for the latter. The explicit shifts are from E[*D*_iso_] = 0.33·10^−9^ m^2^s^−1^ and E[*D*_Δ_²] = 0.07 at 35 Hz to E[*D*_iso_] = 0.37·10^−9^ m^2^s^−1^ and E[*D*_Δ_²] = 0.05 at 320 Hz for the cortex and from E[*D*_iso_] = 0.28·10^−9^ m^2^s^−1^ and E[*D*_Δ_²] = 0.16 at 35 Hz to E[*D*_iso_] = 0.58·10^−9^ m^2^s^−1^ and E[*D*_Δ_²] = 0.03 at 320 Hz for the cerebellum. The *ω*-dependence of E[*D*_iso_] can be reproduced with the closed spherical compartment model (Stepišnik, 1993) using 0.4·10^−9^ m^2^s^−1^ local diffusivity and 4 μm radius for the cortex and 0.7·10^−9^ m^2^s^−1^ local diffusivity and 5 μm radius for the cerebellum. Among many other possible mechanisms, these lower and higher values of the local diffusivity could result from the biologically plausible macromolecular contents of, respectively, 30 and 10 vol% (Topgaard, 2020) in solutions with salt and metabolites reducing the diffusivity to 50% of the pure water reference state. As stated above, this model-based interpretation is certainly oversimplified but here serves the purpose to illustrate that rather subtle differences in local chemical composition or biomembrane geometry may have a large impact on data acquired under conditions that are determined more by hardware constraints than by the wishes of the experimentalist.

**Fig. 3.**
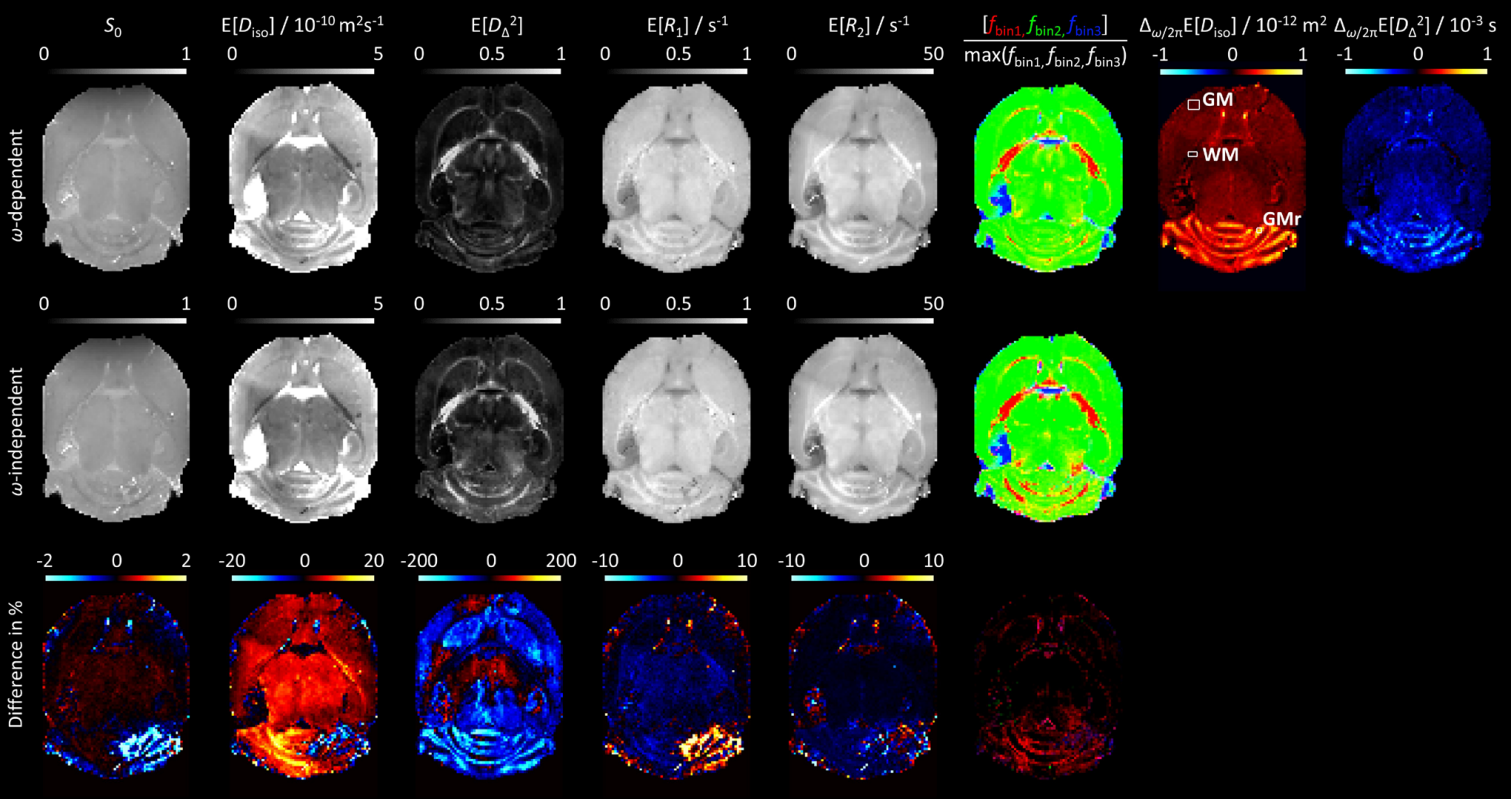
Ex vivo mouse brain parameter maps obtained from *ω*-dependent and *ω*-independent inversions of data acquired with the preclinical (wide *ω*_cent_-range) protocol in Figure 1c. The primary distributions were converted to extrapolated signals *S*_0_, means E[*X*], and bin-resolved signal fractions *f*_bin_*_n_* via Eqs. (17), (16), and (19), respectively, using the frequency *ω*_50%_/2π = 190 Hz labeled in Figure 1f for the *ω*-dependent case. The color coding of *f*_bin_*_n_* is given in Eq. (18). The *ω*-dependence metrics Δ*_ω_*_/2π_E[*X*], defined in Eq. (20), were evaluated using the frequencies *ω*_10%_/2π = 35 Hz and *ω*_90%_/2π = 320 Hz shown in Figure 1f. The normalized differences were calculated according to Eq. (21).

If **D**(*ω*) depends on *ω* within the investigated window, the *ω*-dependent and *ω*-independent analyses give different residuals as well as 2D *D*_iso_-*D*_Δ_² projections. The difference for the latter is minimized if the *ω*-dependent projection is evaluated at the center of the investigated frequency range as quantified by the *ω*_50%_ metric shown in Figure 1f. Visual inspection of the residuals and 2D *D*_iso_-*D*_Δ_² projections in Figure 2 reveal a correlation between misfit and bias toward higher values of *D*_Δ_² with only minor influence on *D*_iso_. The magnitude of the bias is investigated further in Figure 3 showing parameter maps extracted from the distributions obtained by the *ω*-dependent (top row) and *ω*-independent (middle row) analyses, the former being evaluated at *ω*_50%_/2π = 190 Hz. The *ω*-dependence as such is reported in terms of the model-independent dispersion metrics Δ*_ω_*_/2π_E[*D*_iso_] and Δ*_ω_*_/2π_E[*D*_Δ_²] defined in Eq. (20). Superficially, the two analysis approaches appear to give similar parameter maps, except for E[*D*_Δ_²] with noticeably lower values in GM for the *ω*-dependent analysis. For the cerebellum, this effect leads to sharper differentiation between GM and WM. The bias in E[*D*_Δ_²] is mirrored in the bin-resolved signal fractions map, showing lower values of the anisotropic fraction (bin1) in the *ω*-dependent results. The minor differences between the maps are amplified in the normalized difference maps (bottom row) at the expense of exaggerating the deviations when the metrics are near zero. In general, the difference maps are positive (+10%) for E[*D*_iso_], negative (–100%) for E[*D*_Δ_²], and close to zero for *S*_0_, E[*R*_1_], and E[*R*_2_]. An exception to this general observation is the right part of the cerebellum which seems to be contaminated by an image artifact affecting primarily *S*_0_ and E[*R*_1_]. While *S*_0_, *R*_1_, and *R*_2_ do not have any explicit *ω*-dependence, a poor fit in the diffusion dimensions could introduce a bias also in the other metrics. The areas highlighted in the Δ*_ω_*_/2π_E[*D*_iso_] and Δ*_ω_*_/2π_E[*D*_Δ_²] maps, such as the cerebellar GM and the tip of the lateral ventricles (Aggarwal et al., 2012), coincide with the bias in E[*D*_iso_] and E[*D*_Δ_²].

**Fig. 4.**
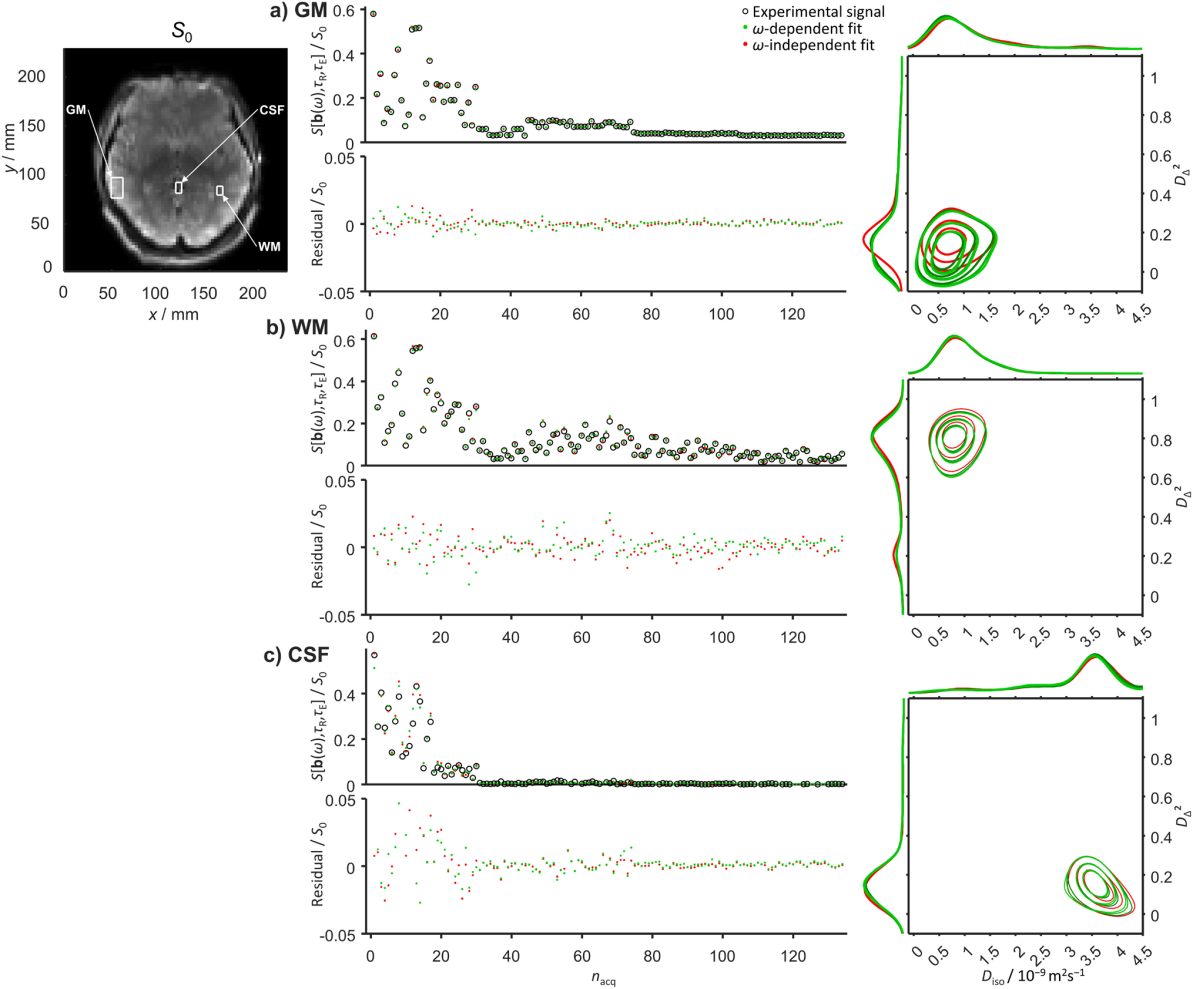
Comparison between *ω*-dependent and *ω*-independent data inversion results for in vivo human brain ROIs (white rectangles) in gray matter (GM), white matter (WM), and cerebrospinal fluid (CSF) displayed over an *S*_0_ map. The data were acquired with the clinical (narrow *ω*_cent_-range) protocol in Figure 1e and the *ω*-dependent 2D *D*_iso_-*D*_Δ_² projections are plotted for three values of *ω* between *ω*_10%_/2π = 5 Hz (dark green) and *ω*_90%_/2π = 11 Hz (pale green). For additional explanations of symbols and labels, see Figure 2 caption.

**Fig. 5.**
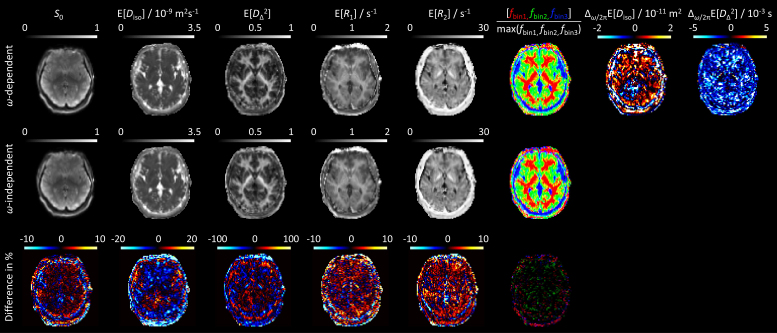
In vivo human brain parameters maps obtained from *ω*-dependent and *ω*-independent inversions of data acquired with the clinical (narrow *ω*-range) protocol in Figure 1e. The *ω*-dependent E[*X*] maps were evaluated at *ω*_50%_/2π = 9 Hz while the *ω*-dependence metrics Δ*_ω_*_/2π_E[*X*] employed *ω*_10%_/2π = 5 Hz and *ω*_90%_/2π = 11 Hz labeled in Figure 1g. For additional explanations, see Figure 3 caption.

Figures 4 and 5 show results for in vivo human brain obtained with the narrow *ω*_cent_-range protocol in Figure 1e. In addition to the numerous exchange and *ω*-dependence mechanisms described above for water in the salt solution, liquid crystal, tumor tissue, and fixated mouse brain, the living brain features processes originating from the beating heart and the varying pressure in the blood vessels. These processes include not only blood flow in the arteries, veins, and capillary network, as well as pulsatile motion of the entire brain (Wagshul et al., 2011), but also flow of cerebrospinal fluid (CSF) in the ventricles, interstitial fluid (ISF) in the extracellular spaces, and mixed CSF and ISF in the perivascular spaces (Jessen et al., 2015), giving rise to dispersion in **D**(*ω*) at frequencies determined by the interplay between the fluid flow rates and vessel curvatures (Callaghan & Stepišnik, 1995). The gradient waveforms in Figure 1d were designed within hardware constraints with the aim of minimizing *τ*_E_ for given values of *b* and *b*_Δ_, unintentionally giving rise to an anisotropic spread of spectral power in **b**(*ω*) for each waveform (Lundell & Lasič, 2020) as well as a variation of *ω*_cent_ from *ω*_10%_/2π = 5 Hz to *ω*_90%_/2π = 11 Hz. Although the relative variation of *ω*_cent_ is sufficient to detect *ω*-dependence as previously demonstrated for ex vivo rat brain (Narvaez et al., 2022, 2024), the data for WM, GM, and CSF rectangle ROIs in Figure 4 show no clear differences between the residuals or 2D *D*_iso_-*D*_Δ_² projections from the *ω*-dependent and *ω*-independent analyses. This absence of *ω*-dependence in the 5–11 Hz range is far from obvious considering the continuous range of structural length scales and dynamical time scales known to exist in the living brain, but is consistent with literature results of identical diffusion tensor distributions at the diffusion times 19 and 49 ms (Song et al., 2022) and the numerous oscillating gradient spin-echo studies finding dispersion predominantly at higher frequencies (Arbabi et al., 2020; Baron & Beaulieu, 2014; Baron et al., 2015; Dai et al., 2023; Hennel et al., 2021; Michael et al., 2022; Tan et al., 2020; Tetreault et al., 2020; Van et al., 2014).

The overall description and interpretation of the in vivo WM and GM data in Figure 4a and b are similar to the ex vivo results above, one minor difference being an oblate component visible in the 1D *D*_Δ_² projection for WM at *D*_Δ_² slightly below 0.25. Spurious oblate components in general appear as inversion artifacts at low signal-to-noise ratio and insufficient exploration of the *b*_Δ_ acquisition dimension (de Almeida Martins & Topgaard, 2018). For acquisition protocols limited to *b*_Δ_ = 1, such oblate components may even dominate the distributions for most voxels except CSF and coherently aligned WM (Song et al., 2022). The *ω*-dependent and *ω*-independent 1D *D*_Δ_² projections for GM are not completely overlapping despite the absence of discernible differences in the corresponding residuals. This effect may originate from the correlation between *ω*_cent_ and *b*_Δ_ at the highest values of *b* in the acquisition protocol in Figure 1e and the slightly lower signal intensities observed for *ω*_cent_/2π = 10 Hz and *b*_Δ_ = –0.5 than for *ω*_cent_/2π = 6 Hz and *b*_Δ_ = 1. At constant *b*, the powder-averaged signal would increase with *b*_Δ_^2^ and decrease with *ω*_cent_ for the cases of anisotropy and restriction, respectively (Jiang et al., 2023). In modified versions of the acquisition protocol, disambiguation between the two cases could be achieved by extending the *ω*_cent_ range for the two values of *b*_Δ_ at the expense of increasing *τ*_E_. The CSF data in Figure 4c show elevated residuals at low *b* which by visual inspection of the raw images may be attributed to signal dropouts from pulsation artifacts (Chen et al., 2015). The relative amplitudes of the residuals in Figure 4 are reflected in the values of SNR calculated as described in Eq. (20) in the Methods section, yielding the mean values 53, 69, and 20 for, respectively, the WM, GM, and CSF ROIs. These values correspond to SNR for a hypothetical *b* = 0, *τ*_R_ = ∞, and *τ*_E_ = 0 image (not actually measured) and include the effects of random noise, imaging artifacts, and signal variations from mechanisms such as pulsation that are not captured by the expression in Eq. (1). Because of the poor fit for the CSF ROI, it is unclear whether the obtained distributions with E[*D*_iso_] = 3.5·10^−9^ m^2^s^−1^, approx. 20% above the literature value 3.0·10^−9^ m^2^s^−1^ for pure water at 37ºC (Holz et al., 2000), are influenced by intravoxel CSF flow. The absence of *ω*-dependence within the investigated 5–11 Hz range is further accentuated by the similarities of the *ω*-dependent and *ω*-independent parameter maps and the lack of brain-specific structures in the difference and dispersion metrics maps in Figure 5.

The 1491-volume preclinical and 131-volume clinical **D**(*ω*)-*R*_1_-*R*_2_ protocols illustrate two extremes for investigating the effects of restriction by comparison of fit residuals, 2D *D*_iso_-*D*_Δ_² projections, and parameter maps obtained by *ω*-dependent and *ω*-independent data inversion. While the preclinical protocol shows superior sensitivity to restriction, it may be too exhaustive for implementation in studies of more than a few specimens. Conversely, the clinical protocol has a measurement time commensurate with clinical research studies, however, with a narrow frequency window where the effects of restriction appear to be absent for healthy human brain. Designing a time-efficient acquisition protocol for solving a particular scientific question is a challenging endeavor which we leave for future research focused on specific applications. Nevertheless, the presented protocols are appropriate for pilot studies of a few cases or specimens to determine which combination of dimensions and metrics—for instance the herein included E[*D*_iso_], E[*D*_Δ_²], E[*R*_1_], E[*R*_2_], *f*_bin_*_n_*, Δ*_ω_*_/2π_E[*D*_iso_], and Δ*_ω_*_/2π_E[*D*_Δ_²] parameters, as well as bin-resolved or higher-order statistical descriptors (Narvaez et al., 2022)—that hold greatest promise for, say, distinguishing between tumor grades or detecting neurodegeneration. Based on such initial data, the protocols could be refined to focus the measurements on parts of the multidimensional acquisition parameter space that show greatest raw signal amplitude differences between the cases to be distinguished, which in general leads to highest precision of the most valuable metrics obtained from the data inversion.

## 5 Conclusion

Multidimensional diffusion–relaxation correlation MRI relying on tensor-valued diffusion encoding is associated with a sensitivity to restricted diffusion that depends on numerous factors including the gradient waveform duration, the encoding tensor shape, and—for most non-linear tensor shapes—the spatial direction. Monte Carlo inversion of such data can be augmented with explicit consideration of the effects of restriction in terms of the frequency-dependence of the tensor-valued diffusion spectrum for acquisition protocols exploring both wide and narrow frequency ranges and samples with and without observable restriction effects within the investigated frequency window. In the former case, inversion including frequency-dependence gives smaller fit residuals, mitigates bias in mainly the anisotropy metrics, and gives anatomically plausible restriction maps. In the latter case, inversions with and without consideration of frequency-dependence give similar fit residuals and maps of parameters unrelated to restriction, but gives frequency-dependence maps that mainly contain inversion noise and non-anatomical structures from image artifacts. Although the local diffusivities of tissue water, the membrane permeabilities, and the structural length scales of the healthy human brain are such that no frequency-dependence can be observed within the narrow 5–11 Hz window easily accessible with tensor-valued encoding on clinical scanners, the situation may be different for pathological tissues or protocols optimized for expanded frequency range. Consequently, we propose that frequency-dependence is included in the data inversion by default to reduce bias in the diffusion metrics and allow detection of restriction in the somewhat unpredictable cases where its effect on the raw signal intensities exceeds the ones of the ever-present image artifacts.

## Supplementary Material

Supplementary Material

## Figures and Tables

**Appendix Fig. A1.**
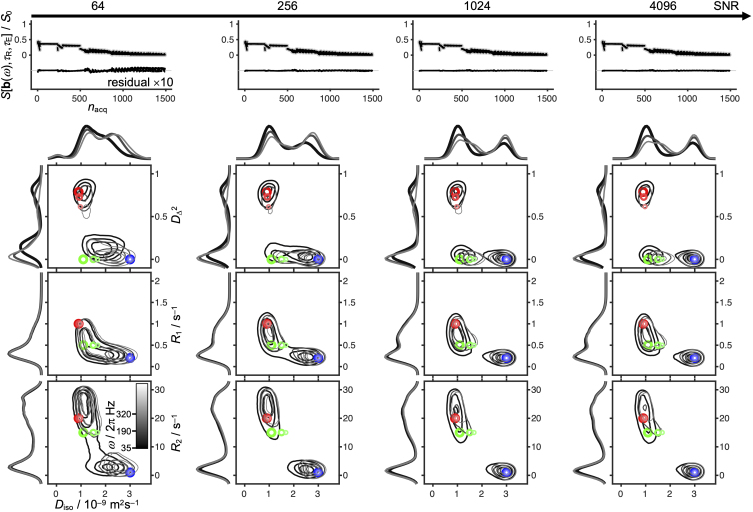
Signal data *S*[**b**(*ω*),*τ*_R_,*τ*_E_] (top row) and projections of the **D**(*ω*)-*R*_1_-*R*_2_ distributions (bottom rows) for the preclinical protocol in Figure 1c at four values of the SNR (columns). Noise-free ground-truth and back-calculated signals are shown with gray circles and black dots, respectively, as a function of *n*_acq_. Ground-truth values of *D*_iso_, *D*_Δ_^2^, *R*_1_, and *R*_2_, for the WM- (red), GM- (green), and CSF-like (blue) components are shown as progressively smaller and brighter circles at values of *ω*/2π corresponding to *ω*_10%_/2π = 35 Hz, *ω*_50%_/2π = 190 Hz, and *ω*_90%_/2π = 320 Hz of the acquisition protocol. 2D contours and 1D traces (lines with gray scale given by *ω*/2π) represent projections of the distributions obtained by Monte Carlo inversion of the signal data.

**Appendix Fig. A2.**
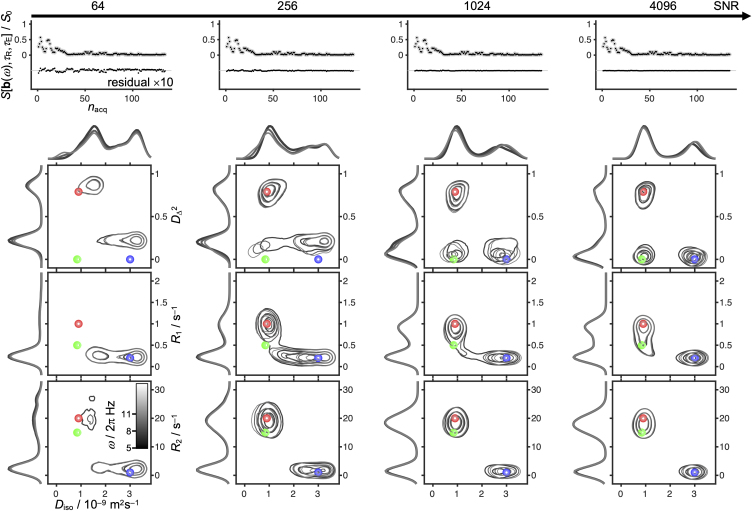
Signal data and distributions for the clinical protocol in Figure 1e with *ω*_10%_/2π = 5 Hz, *ω*_50%_/2π = 8 Hz, and *ω*_90%_/2π = 11 Hz. See Appendix Figure A1 caption for additional details.

## Data Availability

MATLAB source code for Monte-Carlo data inversion is freely available at https://github.com/daniel-topgaard/md-dmri/.

## Appendix: Monte Carlo Inversion of Simulated Data

### A.1 Generation of signal data and distributions

Signal data *S*[**b**(*ω*),*τ*_R_,*τ*_E_] were synthesized using the preclinical and clinical acquisition protocols in Figure 1 for a single voxel containing equal signal contributions from three distinct components roughly corresponding to WM, GM, and CSF for *in vivo* human brain. For the WM-like component, **D**(*ω*) was expressed as an axisymmetric tensor, according to Eq. (11), with axial diffusivity *D*_A_ equal to the bulk liquid (high-*ω*) diffusivity *D*_0_ and an *ω*-dependent radial diffusivity *D*_R_(*ω*) given by the cylinder model (Stepišnik, 1993; Stepišnik et al., 2006)

$$D_{\text{R}}\left(\omega \right)=D_{0}-\sum_{k=1}^{\infty }w_{k}\frac{D_{0}-D_{\infty }}{1+\omega^{2}\text{​}/\Gamma_{k}^{2}},$$
(A1)

where

$$w_{k}=\frac{2}{\zeta_{k}^{2}-1},$$
(A2)

and

$$\Gamma_{k}=\frac{\zeta_{k}^{2}D_{0}}{r^{2}}.$$
(A3)

In the equations above, *D*_∞_ is the tortuosity-limit (low-*ω*) diffusivity, *r* the cylinder radius, and *ζ_k_* the *k*th root of

$$\zeta J_{0}\left(\zeta \right)-J_{1}\left(\zeta \right)=0,$$
(A4)

where *J_ν_* is the *ν*th order Bessel function of the first kind. The GM-like component was described with an isotropic **D**(*ω*) with all eigenvalues *D*(*ω*) given by the random permeable barrier model (Novikov et al., 2011)

$$D\left(\omega \right)=\frac{D_{0}}{\frac{D_{0}}{D_{\infty }}+2z_{\omega }\left(1-z_{\omega }\right)\left[\sqrt{1+\frac{D_{0}/D_{\infty }-1}{{\left(1-z_{\omega }\right)}^{2}}}-1\right]},$$
(A5)

where

$$z_{\omega }=\text{i}\sqrt{\text{i}\omega /\Gamma }\;$$
(A6)

and Γ is the characteristic rate for the transition between the high- and low-*ω* plateau values *D*_0_ and *D*_∞_. The CSF-like component was given by an isotropic and *ω*-independent tensor with all eigenvalues equal to *D*_0_. Both the cylinder and random permeable barrier models in Eqs. (A1) and (A5), respectively, rely on the powerful Gaussian phase distribution approximation (Neuman, 1974) that enables prediction of the signal for quite general gradient waveforms, such as the ones in Figure 1b and d, but fails to account for diffraction-like effects that may occur under the special condition of diffusion encoding by pairs of narrow gradient pulses separated by a time period of sufficient duration to give molecular displacements across an isolated compartment or between the centers of neighboring compartments in a periodic geometry (Callaghan et al., 1991). The validity of the approximation has been investigated with random walk simulations for diffusion encoding by gradient pulse pairs, cos-modulated oscillating gradients, and double rotation waveforms (Balinov et al., 1993; Topgaard, 2025). Custom pulse sequences for detecting deviations from the Gaussian phase distribution approximation have been demonstrated for in vivo rat (Henriques et al., 2020) and human (Novello et al., 2022) brain. Reconciling the design constraints of these sequences with the numerically optimized waveforms for efficient tensor-valued encoding in vivo remains a challenge.

Diffusion-related parameters as listed in Appendix Table A1 were used to calculate **D**(*ω*) according to Eqs. (A1)–(A6). The values of *D*_0_, *D*_∞_, *r*, and Γ were selected to emulate *D*_iso_ and *D*_Δ_^2^ observed with the clinical protocol (5–11 Hz), as well as to give pronounced *ω*-dependence with the preclinical (35–320 Hz) protocol. Together with the signal weights and relaxation rates in Appendix Table A1, the calculated **D**(*ω*) was inserted into Eq. (1) to generate ground-truth *S*[**b**(*ω*),*τ*_R_,*τ*_E_].

**Appendix Table A1.** Weights, relaxation rates, and diffusion parameters for generating signal data in Appendix Figures A1 and A2.

component	*w*	*R*_1 _/s^–1^	*R*_2_ /s^–1^	geometry	*D*_0 _/10^–9^ m^2^s^–1^	*D*_∞_ /10^–9^ m^2^s^–1^	transition	orientation
WM	1/3	1	20	cylinder	2.5	0.10	*r* = 1.0 μm	*ϕ* = 20º, *θ* = –30º
GM	1/3	0.5	15	RPBM^*^	2.5	0.70	Γ = 100 s^–1^	
CSF	1/3	0.2	1	free	3.0			

* RPBM: random permeable barrier model.

For both acquisition protocols, Monte Carlo data inversion was performed as described in the main text Methods section using the settings for the clinical protocol with the exceptions of the number of output components and bootstrapping repetitions, which were increased to 50 and 1000, respectively. Additionally, instead of random sampling with replacement, bootstrapping employed the same ground-truth data using different realizations of Gaussian noise with signal-to-noise ratios SNR = 64, 256, 1024, and 4096 at the signal levels corresponding to the *S*_0_ data, where *b* = 0, *τ*_R_ = ∞, and *τ*_E_ = 0. At the minimum values of *τ*_E_ included in the protocols, the SNR levels are approximately a factor of 2 lower.

### A.2 Effect of noise on resolution of distribution components

Signal data and inversion results are shown in Appendix Figures A1 and A2 for the preclinical and clinical protocols, respectively. At the highest SNR, the back-calculated signals and the major peaks in the 2D projections of the distributions reproduce the ground truth. The widths of these peaks reflect the numerical instability of the data inversion as well as the finite number of sharp Lorentzian transitions required to reproduce the smoother transitions given by the geometrical models in Eqs. (A1) and (A5). Noteworthily, the *ω*-dependencies of the WM- and GM-like components, as here represented by the cylinder and random permeable barrier models, are simultaneously detected in the preclinical (35–320 Hz) protocol, but are too small to observe with the clinical (5–11 Hz) one. Since the underlying *ω*-dependence is the same for both cases, these results illustrate that the numerical values of restriction metrics and even microstructural conclusions—for instance, isolated compartments versus structural disorder—may be worryingly sensitive to the specifics of the acquisition protocol. Decreasing SNR leads to increasing fit residuals and loss of resolution of the three components in the 2D projections. At the SNR levels typical for in vivo conditions, it is not reasonable to expect multimodal distributions to be faithfully reproduced in the 2D projections. Still, the results may be converted to lower-level statistical descriptors, such as the means E[*X*] displayed as parameter maps in Figures 3 and 5, as well as variances V[*X*] and covariances C[*X*,*Y*] condensing the information about intra-voxel heterogeneity into scalar metrics that are less susceptible to measurement noise (de Almeida Martins et al., 2020). For the special case of a clinically feasible protocol using 85 acquisitions with varying *b*, *b*_Δ_, Θ, and Φ at narrow range of *ω* and constant *τ*_R_ and *τ*_E_, the accuracy and precision of metrics equivalent to E[*D*_iso_], E[*D*_Δ_^2^], and V[*D*_iso_] were previously investigated for data synthesized from a wide range of ground-truth distributions at infinite and typical in vivo SNR (Reymbaut et al., 2020a). Extending this comprehensive uncertainty analysis to the numerous metrics and relevant distribution dimensions offered by the additional variation of *ω*, *τ*_R_, and *τ*_E_ is, however, beyond the scope of the present work.

## References

[IMAG.a.143-b1] Aggarwal, M., Burnsed, J., Martin, L. J., Northington, F. J., & Zhang, J. (2014). Imaging neurodegeneration in the mouse hippocampus after neonatal hypoxia-ischemia using oscillating gradient diffusion MRI. Magn Reson Med, 72, 829–840. 10.1002/mrm.24956 24123409 PMC3976472

[IMAG.a.143-b2] Aggarwal, M., Jones, M. V., Calabresi, P. A., Mori, S., & Zhang, J. (2012). Probing mouse brain microstructure using oscillating gradient diffusion MRI. Magn Reson Med, 67, 98–109. 10.1002/mrm.22981 21590726 PMC3162097

[IMAG.a.143-b3] Aranda, R., Ramirez-Manzanares, A., & Rivera, M. (2015). Sparse and adaptive diffusion dictionary (SADD) for recovering intra-voxel white matter structure. Med Image Anal, 26, 243–255. 10.1016/j.media.2015.10.002 26519793

[IMAG.a.143-b4] Arbabi, A., Kai, J., Khan, A. R., & Baron, C. A. (2020). Diffusion dispersion imaging: Mapping oscillating gradient spin-echo frequency dependence in the human brain. Magn Reson Med, 83, 2197–2208. 10.1002/mrm.28083 31762110

[IMAG.a.143-b5] Assaf, Y., & Basser, P. J. (2005). Composite hindered and restricted model of diffusion (CHARMED) MR imaging of the human brain. Neuroimage, 27, 48–58. 10.1016/j.neuroimage.2005.03.042 15979342

[IMAG.a.143-b6] Balinov, B., Jönsson, B., Linse, P., & Söderman, O. (1993). The NMR self-diffusion method applied to restricted diffusion. Simulation of echo attenuation from molecules in spheres and between planes. J Magn Reson A, 104, 17–25. 10.1006/jmra.1993.1184

[IMAG.a.143-b7] Balucani, U., Brodholt, J. P., & Vallauri, R. (1996). Analysis of the velocity autocorrelation function of water. J Phys Condens Matter, 8, 6139–6144. 10.1088/0953-8984/8/34/004

[IMAG.a.143-b8] Banani, S. F., Lee, H. O., Hyman, A. A., & Rosen, M. K. (2017). Biomolecular condensates: Organizers of cellular biochemistry. Nat Rev Mol Cell Biol, 18, 285–298. 10.1038/nrm.2017.7 28225081 PMC7434221

[IMAG.a.143-b9] Baron, C. A., & Beaulieu, C. (2014). Oscillating gradient spin-echo (OGSE) diffusion tensor imaging of the human brain. Magn Reson Med, 72, 726–736. 10.1002/mrm.24987 24142863

[IMAG.a.143-b10] Baron, C. A., Kate, M., Gioia, L., Butcher, K., Emery, D., Budde, M., & Beaulieu, C. (2015). Reduction of diffusion-weighted imaging contrast of acute ischemic stroke at short diffusion times. Stroke, 46, 2136–2141. 10.1161/STROKEAHA.115.008815 26152297

[IMAG.a.143-b11] Basser, P. J., Mattiello, J., & Le Bihan, D. (1994). Estimation of the effective self-diffusion tensor from the NMR spin echo. J Magn Reson B, 103, 247–254. 10.1006/jmrb.1994.1037 8019776

[IMAG.a.143-b12] Basser, P. J., & Pajevic, S. (2003). A normal distribution for tensor-valued random variables: Applications to diffusion tensor MRI. IEEE Trans Med Imaging, 22, 785–794. 10.1109/TMI.2003.815059 12906233

[IMAG.a.143-b13] Beaulieu, C. (2002). The basis of anisotropic water diffusion in the nervous system—A technical review. NMR Biomed, 15, 435–455. 10.1002/nbm.782 12489094

[IMAG.a.143-b14] Ben-Shaul, A., & Gelbart, W. M. (1985). Theory of chain packing in amphiphilic aggregates. Ann Rev Phys Chem, 36, 179–211. 10.1146/annurev.pc.36.100185.001143

[IMAG.a.143-b15] Benjamini, D. (2020). Nonparametric inversion of relaxation and diffusion correlation data. In D. Topgaard (Ed.), Advanced diffusion encoding methods in MRI (pp. 278–316). Royal Society of Chemistry. 10.1039/9781788019910-00278

[IMAG.a.143-b16] Benjamini, D., & Basser, P. J. (2020). Multidimensional correlation MRI. NMR Biomed, 33(12), e4226. 10.1002/nbm.4226 31909516 PMC11062766

[IMAG.a.143-b17] Berman, P., Levi, O., Parmet, Y., Saunders, M., & Wiesman, Z. (2013). Laplace inversion of low-resolution NMR relaxometry data using sparse representation methods. Conc Magn Reson A, 42, 72–88. 10.1002/cmr.a.21263 PMC3698697 23847452

[IMAG.a.143-b18] Bernin, D., Koch, V., Nydén, M., & Topgaard, D. (2014). Multi-scale characterization of lyotropic liquid crystals using ^2^H and diffusion MRI with spatial resolution in three dimensions. PLoS One, 9, e98752. 10.1371/journal.pone.0098752 24905818 PMC4048170

[IMAG.a.143-b19] Bernin, D., & Topgaard, D. (2013). NMR diffusion and relaxation correlation methods: New insights in heterogeneous materials. Curr Opin Colloid Interface Sci, 18, 166–172. 10.1016/j.cocis.2013.03.007

[IMAG.a.143-b20] Bleuzen, A., Pittet, P.-A., Helm, L., & Merbach, A. E. (1997). Water exchange on magnesium(II) in aqueous solution: A variable temperature and pressure ^17^O NMR study. Magn Reson Chem, 35, 765–773. 10.1002/(sici)1097-458x(199711)35:11<765::Aid-omr169>3.0.Co;2-f

[IMAG.a.143-b21] Boss, B. D., & Stejskal, E. O. (1965). Anisotropic diffusion in hydrated vermiculite. J Chem Phys, 43, 1068–1069. 10.1063/1.1696823

[IMAG.a.143-b22] Callaghan, P. T., Coy, A., MacGowan, D., Packer, K. J., & Zelaya, F. O. (1991). Diffraction-like effects in NMR diffusion studies of fluids in porous solids. Nature, 351, 467–469. 10.1038/351467a0

[IMAG.a.143-b23] Callaghan, P. T., & Stepišnik, J. (1995). Frequency-domain analysis of spin motion using modulated-gradient NMR. J Magn Reson A, 117, 118–122. 10.1006/jmra.1995.9959

[IMAG.a.143-b24] Chen, L., Beckett, A., Verma, A., & Feinberg, D. A. (2015). Dynamics of respiratory and cardiac CSF motion revealed with real-time simultaneous multi-slice EPI velocity phase contrast imaging. Neuroimage, 122, 281–287. 10.1016/j.neuroimage.2015.07.073 26241682 PMC4716739

[IMAG.a.143-b25] Clark, C. A., Hedehus, M., & Moseley, M. E. (2001). Diffusion time dependence of the apparent diffusion tensor in healthy human brain and white matter disease. Magn Reson Med, 45, 1126–1129. 10.1002/mrm.1149 11378893

[IMAG.a.143-b26] Colvin, D. C., Loveless, M. E., Does, M. D., Yue, Z., Yankeelov, T. E., & Gore, J. C. (2011). Earlier detection of tumor treatment response using magnetic resonance diffusion imaging with oscillating gradients. Magn Reson Imaging, 29, 315–323. 10.1016/j.mri.2010.10.003 21190804 PMC3285502

[IMAG.a.143-b27] Colvin, D. C., Yankeelov, T. E., Does, M. D., Yue, Z., Quarles, C., & Gore, J. C. (2008). New insights into tumor microstructure using temporal diffusion spectroscopy. Cancer Res, 68, 5941–5947. 10.1158/0008-5472.CAN-08-0832 18632649 PMC2719758

[IMAG.a.143-b28] Conturo, T. E., McKinstry, R. C., Akbudak, E., & Robinson, B. H. (1996). Encoding of anisotropic diffusion with tetrahedral gradients: A general mathematical diffusion formalism and experimental results. Magn Reson Med, 35, 399–412. 10.1002/mrm.1910350319 8699953

[IMAG.a.143-b29] Cooper, R. L., Chang, D. B., Young, A. C., Martin, C. J., & Ancker-Johnson, B. (1974). Restricted diffusion in biophysical systems: Experiment. Biophys J, 14, 161–177. 10.1016/S0006-3495(74)85904-7 4823458 PMC1334492

[IMAG.a.143-b30] Cordero-Grande, L., Christiaens, D., Hutter, J., Price, A. N., & Hajnal, J. V. (2019). Complex diffusion-weighted image estimation via matrix recovery under general noise models. Neuroimage, 200, 391–404. 10.1016/j.neuroimage.2019.06.039 31226495 PMC6711461

[IMAG.a.143-b31] Dai, E., Zhu, A., Yang, G. K., Quah, K., Tan, E. T., Fiveland, E., Foo, T. K. F., & McNab, J. A. (2023). Frequency-dependent diffusion kurtosis imaging in the human brain using an oscillating gradient spin echo sequence and a high-performance head-only gradient. Neuroimage, 279, 120328. 10.1016/j.neuroimage.2023.120328 37586445 PMC10529993

[IMAG.a.143-b32] Daimiel Naranjo, I., Reymbaut, A., Brynolfsson, P., Lo Gullo, R., Bryskhe, K., Topgaard, D., Giri, D. D., Reiner, J. S., Thakur, S., & Pinker-Domenig, K. (2021). Multidimensional diffusion magnetic resonance imaging for characterization of tissue microstructure in breast cancer patients: A prospective pilot study. Cancers, 13, 1606. 10.3390/cancers13071606 33807205 PMC8037718

[IMAG.a.143-b33] de Almeida Martins, J. P., Tax, C. M. W., Reymbaut, A., Szczepankiewicz, F., Chamberland, M., Jones, D. K., & Topgaard, D. (2021). Computing and visualising intra-voxel orientation-specific relaxation-diffusion features in the human brain. Hum Brain Mapp, 42, 310–328. 10.1002/hbm.25224 33022844 PMC7776010

[IMAG.a.143-b34] de Almeida Martins, J. P., Tax, C. M. W., Szczepankiewicz, F., Jones, D. K., Westin, C.-F., & Topgaard, D. (2020). Transferring principles of solid-state and Laplace NMR to the field of in vivo brain MRI. Magn Reson, 1, 27–43. 10.5194/mr-1-27-2020 PMC10500744 37904884

[IMAG.a.143-b35] de Almeida Martins, J. P., & Topgaard, D. (2016). Two-dimensional correlation of isotropic and directional diffusion using NMR. Phys Rev Lett, 116, 087601. 10.1103/PhysRevLett.116.087601 26967442

[IMAG.a.143-b36] de Almeida Martins, J. P., & Topgaard, D. (2018). Multidimensional correlation of nuclear relaxation rates and diffusion tensors for model-free investigations of heterogeneous anisotropic porous materials. Sci Rep, 8, 2488. 10.1038/s41598-018-19826-9 29410433 PMC5802831

[IMAG.a.143-b37] de Swiet, T. M., & Mitra, P. P. (1996). Possible systematic errors in single-shot measurements of the trace of the diffusion tensor. J Magn Reson B, 111, 15–22. 10.1006/jmrb.1996.0055 8661259

[IMAG.a.143-b38] Does, M. D., Parsons, E. C., & Gore, J. C. (2003). Oscillating gradient measurements of water diffusion in normal and globally ischemic rat brain. Magn Reson Med, 49, 206–215. 10.1002/mrm.10385 12541239

[IMAG.a.143-b39] Edelstein, W. A., Hutchison, J. M. S., Johnson, G., & Redpath, T. (1980). Spin warp NMR imaging and applications to human whole-body imaging. Phys Med Biol, 25, 751–756. 10.1088/0031-9155/25/4/017 7454767

[IMAG.a.143-b40] Edzes, H. T., & Samulski, E. T. (1975). Cross relaxation and spin diffusion in the proton NMR of hydrated collagen. Nature, 265, 521–523. 10.1038/265521a0 834303

[IMAG.a.143-b41] Ekwall, P., Mandell, L., & Fontell, K. (1969). Ternary systems of potassium soap, alcohol, and water. J Colloid Interface Sci, 31, 508–529. 10.1016/0021-9797(69)90052-6 5788924

[IMAG.a.143-b42] Eriksson, S., Lasič, S., Nilsson, M., Westin, C.-F., & Topgaard, D. (2015). NMR diffusion encoding with axial symmetry and variable anisotropy: Distinguishing between prolate and oblate microscopic diffusion tensors with unknown orientation distribution. J Chem Phys, 142, 104201. 10.1063/1.4913502 25770532 PMC4359170

[IMAG.a.143-b43] Eriksson, S., Lasič, S., & Topgaard, D. (2013). Isotropic diffusion weighting by magic-angle spinning of the *q*-vector in PGSE NMR. J Magn Reson, 226, 13–18. 10.1016/j.jmr.2012.10.015 23178533

[IMAG.a.143-b44] Evans, D. F., & Wennerström, H. (1999). The colloidal domain: Where physics, chemistry, biology, and technology meet (2nd ed.). Wiley-VCH. https://www.wiley.com/en-us/The+Colloidal+Domain%3A+Where+Physics%2C+Chemistry%2C+Biology%2C+and+Technology+Meet%2C+2nd+Edition-p-9780471242475

[IMAG.a.143-b45] Galvosas, P., & Callaghan, P. T. (2010). Multi-dimensional inverse Laplace spectroscopy in the NMR of porous media. C R Phys, 11, 172–180. 10.1016/j.crhy.2010.06.014

[IMAG.a.143-b46] Helm, L., & Merbach, A. E. (1999). Water exchange on metal ions: Experiments and simulations. Coord Chem Rev, 187, 151–181. 10.1016/S0010-8545(99)90232-1

[IMAG.a.143-b47] Hennel, F., Michael, E. S., & Pruessmann, K. P. (2021). Improved gradient waveforms for oscillating gradient spin-echo (OGSE) diffusion tensor imaging. NMR Biomed, 34, e4434. 10.1002/nbm.4434 33124071

[IMAG.a.143-b48] Hennig, J., Nauerth, A., & Friedurg, H. (1986). RARE imaging: A fast imaging method for clinical MR. Magn Reson Med, 3, 823–833. 10.1002/mrm.1910030602 3821461

[IMAG.a.143-b49] Henriques, R. N., Jespersen, S. N., & Shemesh, N. (2020). Correlation tensor magnetic resonance imaging. Neuroimage, 211, 116605. 10.1016/j.neuroimage.2020.116605 32044435

[IMAG.a.143-b50] Hills, B. P. (1990). Nuclear magnetic resonance relaxation studies of proton exchange in methanol-water mixtures. J Chem Soc Faraday Trans, 86, 481–487. 10.1039/FT9908600481

[IMAG.a.143-b51] Hills, B. P., Wright, K. M., & Belton, P. S. (1989). Proton N.M.R. studies of chemical and diffusive exchange in carbohydrate systems. Mol Phys, 67, 1309–1326. 10.1080/00268978900101831

[IMAG.a.143-b52] Holz, M., Heil, S. R., & Sacco, A. (2000). Temperature-dependent self-diffusion coefficients of water and six selected molecular liquids for calibration in accurate ^1^H NMR PFG measurements. Phys Chem Chem Phys, 2, 4740–4742. 10.1039/b005319h

[IMAG.a.143-b53] Hyman, A. A., Weber, C. A., & Jülicher, F. (2014). Liquid-liquid phase separation in biology. Annu Rev Cell Dev Biol, 30, 39–58. 10.1146/annurev-cellbio-100913-013325 25288112

[IMAG.a.143-b54] Jespersen, S. N., Olesen, J. L., Ianus, A., & Shemesh, N. (2019). Effects of nongaussian diffusion on “isotropic diffusion” measurements: An ex-vivo microimaging and simulation study. J Magn Reson, 300, 84–94. 10.1016/j.jmr.2019.01.007 30711786

[IMAG.a.143-b55] Jessen, N. A., Munk, A. S., Lundgaard, I., & Nedergaard, M. (2015). The glymphatic system: A beginner’s guide. Neurochem Res, 40, 2583–2599. 10.1007/s11064-015-1581-6 25947369 PMC4636982

[IMAG.a.143-b56] Jian, B., Vemuri, B. C., Özarslan, E., Carney, P. R., & Mareci, T. H. (2007). A novel tensor distribution model for the diffusion-weighted MR signal. Neuroimage, 37, 164–176. 10.1016/j.neuroimage.2007.03.074 17570683 PMC2576290

[IMAG.a.143-b57] Jiang, H., de Almeida Martins, J. P., Lundberg, D., Tax, C. M. W., & Topgaard, D. (2021). Lamellar liquid crystal phantom for validating MRI methods to distinguish oblate and prolate diffusion tensors on whole-body scanners. Proc Intl Soc Mag Reson Med, 29, 3417.

[IMAG.a.143-b58] Jiang, H., Svenningsson, L., & Topgaard, D. (2023). Multidimensional encoding of restricted and anisotropic diffusion by double rotation of the q vector. Magn Reson, 4, 73–85. 10.5194/mr-4-73-2023 PMC10583292 37904800

[IMAG.a.143-b59] Johnson Jr, C. S. (1993). Effects of chemical exchange in diffusion-ordered 2D NMR spectra. J Magn Reson A, 102, 214–218. 10.1006/jmra.1993.1093

[IMAG.a.143-b60] Johnson, J. T. E., Irfanoglu, M. O., Manninen, E., Ross, T. J., Yang, Y., Laun, F. B., Martin, J., Topgaard, D., & Benjamini, D. (2024). In vivo disentanglement of diffusion frequency‐dependence, tensor shape, and relaxation using multidimensional MRI. Hum Brain Mapp, 45, e26697. 10.1002/hbm.26697 38726888 PMC11082920

[IMAG.a.143-b61] Jones, D. K. (Ed.). (2010). Diffusion MRI: Theory, methods, and applications. Oxford University Press https://academic.oup.com/book/24921?login=false

[IMAG.a.143-b62] Jost, W. (1952). Diffusion in solids, liquids, and gases. Academic Press. https://onlinelibrary.wiley.com/doi/abs/10.1002/ange.19530651912

[IMAG.a.143-b63] Kärger, J. (1969). *Zur Bestimmung der Diffusion in einem Zweibereichsystem mit Hilfe von gepulsten Feldgradienten*. Ann Phys, 479, 1–4. 10.1002/andp.19694790102

[IMAG.a.143-b64] Kellner, E., Dhital, B., Kiselev, V. G., & Reisert, M. (2016). Gibbs-ringing artifact removal based on local subvoxel-shifts. Magn Reson Med, 76, 1574–1581. 10.1002/mrm.26054 26745823

[IMAG.a.143-b65] Kingsley, P. B. (2006). Introduction to diffusion tensor imaging mathematics: Part II. Anisotropy, diffusion-weighting factors, and gradient encoding schemes. Conc Magn Reson A, 28A, 123–154. 10.1002/cmr.a.20049

[IMAG.a.143-b66] Klein, S., Staring, M., Murphy, K., Viergever, M. A., & Pluim, J. P. (2010). elastix: A toolbox for intensity-based medical image registration. IEEE Trans Med Imaging, 29, 196–205. 10.1109/TMI.2009.2035616 19923044

[IMAG.a.143-b67] Koay, C. G., & Basser, P. J. (2006). Analytically exact correction scheme for signal extraction from noisy magnitude MR signals. J Magn Reson, 179, 317–322. 10.1016/j.jmr.2006.01.016 16488635

[IMAG.a.143-b68] Lasič, S., Szczepankiewicz, F., Eriksson, S., Nilsson, M., & Topgaard, D. (2014). Microanisotropy imaging: Quantification of microscopic diffusion anisotropy and orientational order parameter by diffusion MRI with magic-angle spinning of the *q*-vector. Front Physics, 2, 11. 10.3389/fphy.2014.00011

[IMAG.a.143-b69] Latour, L. L., Kleinberg, R. L., Mitra, P. P., & Sotak, C. H. (1995). Pore-size distributions and tortuosity in heterogeneous porous media. J Magn Reson A, 112, 83–91. 10.1006/jmra.1995.1012

[IMAG.a.143-b70] Latour, L. L., Mitra, P. P., Kleinberg, R. L., & Sotak, C. H. (1993). Time-dependent diffusion coefficient of fluids in porous media as a probe of surface-to-volume ratio. J Magn Reson A, 101, 342–346. 10.1006/jmra.1993.1056

[IMAG.a.143-b71] Latour, L. L., Svoboda, K., Mitra, P. P., & Sotak, C. H. (1994). Time-dependent diffusion of water in a biological model system. Proc Natl Acad Sci USA, 91, 1229–1233. 10.1073/pnas.91.4.1229 8108392 PMC43130

[IMAG.a.143-b72] Laukien, G., & Schlüter, J. (1956). Impulstechnische Messungen der Spin-Gitter und der Spin-Spin-Relaxationszeiten von Protonen in wäßrigen Lösungen paramagnetischer Ionen. Z Physik, 146, 113–126. 10.1007/BF01326008

[IMAG.a.143-b73] Le Bihan, D., Breton, E., Lallemand, D., Grenier, P., Cabanis, E., & Laval-Jeantet, M. (1986). MR imaging of intravoxel incoherent motions—Application to diffusion and perfusion in neurological disorders. Radiology, 161, 401–407. 10.1148/radiology.161.2.3763909 3763909

[IMAG.a.143-b74] Le Bihan, D., Turner, R., & Douek, P. (1993). Is water diffusion restricted in human brain white matter? Neuroreport, 4, 887–890. 10.1097/00001756-199307000-00012 8369479

[IMAG.a.143-b75] Leow, A. D., Zhu, S., Zhan, L., McMahon, K., de Zubicaray, G. I., Meredith, M., Wright, M. J., Toga, A. W., & Thompson, P. M. (2009). The tensor distribution function. Magn Reson Med, 61, 205–214. 10.1002/mrm.21852 19097208 PMC2770429

[IMAG.a.143-b76] Li, H., Gore, J. C., & Xu, J. (2014). Fast and robust measurement of microstructural dimensions using temporal diffusion spectroscopy. J Magn Reson, 242, 4–9. 10.1016/j.jmr.2014.02.007 24583517 PMC4008665

[IMAG.a.143-b77] Ligneul, C., & Valette, J. (2017). Probing metabolite diffusion at ultra-short time scales in the mouse brain using optimized oscillating gradients and “short”-echo-time diffusion-weighted MRS. NMR Biomed, 30, e3671. 10.1002/nbm.3671 PMC5164933 27891691

[IMAG.a.143-b78] Lindahl, E., & Edholm, O. (2000). Mesoscopic undulations and thickness fluctuations in lipid bilayers from molecular dynamics simulations. Biophys J, 79, 426–433. 10.1016/S0006-3495(00)76304-1 10866968 PMC1300946

[IMAG.a.143-b79] Lundell, H., & Lasič, S. (2020). Diffusion encoding with general gradient waveforms. In D. Topgaard (Ed.), Advanced diffusion encoding methods in MRI (pp. 12–67). Royal Society of Chemistry. 10.1039/9781788019910-00012

[IMAG.a.143-b80] Lundell, H., Nilsson, M., Dyrby, T. B., Parker, G. J. M., Cristinacce, P. L. H., Zhou, F. L., Topgaard, D., & Lasič, S. (2019). Multidimensional diffusion MRI with spectrally modulated gradients reveals unprecedented microstructural detail. Sci Rep, 9, 9026. 10.1038/s41598-019-45235-7 31227745 PMC6588609

[IMAG.a.143-b81] Lundell, H., Sønderby, C. K., & Dyrby, T. B. (2015). Diffusion weighted imaging with circularly polarized oscillating gradients. Magn Reson Med, 73, 1171–1176. 10.1002/mrm.25211 24639209

[IMAG.a.143-b82] Lutti, A., & Callaghan, P. T. (2007). Measurement of multilamellar onion dimensions under shear using frequency domain pulsed gradient NMR. J Magn Reson, 187, 251–257. 10.1016/j.jmr.2007.05.003 17533141

[IMAG.a.143-b83] MacGregor, R. P., Peemoeller, H., Schneider, M. H., & Sharp, A. R. (1983). Anisotropic diffusion of water in wood. J Appl Polym Sci Applied Polymer Symposium, 37, 901–909. https://chemport-n.cas.org//chemport-n/?APP=ftslink&action=reflink&origin=npg&version=1.0&coi=1%3ACAS%3A528%3ADyaL2cXmtFarurk%3D&md5=73536d3d2b6fbe713cb13f3f7f274697

[IMAG.a.143-b84] Magdoom, K. N., Pajevic, S., Dario, G., & Basser, P. J. (2021). A new framework for MR diffusion tensor distribution. Sci Rep, 11, 2766. 10.1038/s41598-021-81264-x 33531530 PMC7854653

[IMAG.a.143-b85] Manninen, E., Bao, S., Landman, B. A., Yang, Y., Topgaard, D., & Benjamini, D. (2024). Variability of multidimensional diffusion-relaxation MRI estimates in the human brain. Imaging Neurosci, 2, 1–24. 10.1162/imag_a_00387 PMC12315732 40800299

[IMAG.a.143-b86] Martin, J., Endt, S., Wetscherek, A., Kuder, T. A., Doerfler, A., Uder, M., Hensel, B., & Laun, F. B. (2020). Contrast-to-noise ratio analysis of microscopic diffusion anisotropy indices in q-space trajectory imaging. Z Med Phys, 30, 4–16. 10.1016/j.zemedi.2019.01.003 30853147

[IMAG.a.143-b87] Martin, J., Reymbaut, A., Schmidt, M., Doerfler, A., Uder, M., Laun, F. B., & Topgaard, D. (2021). Nonparametric **D**-*R*_1_-*R*_2_ distribution MRI of the living human brain. Neuroimage, 245, 118753. 10.1016/j.neuroimage.2021.118753 34852278

[IMAG.a.143-b88] Michael, E. S., Hennel, F., & Pruessmann, K. P. (2022). Evaluating diffusion dispersion across an extended range of b-values and frequencies: Exploiting gap-filled OGSE shapes, strong gradients, and spiral readouts. Magn Reson Med, 87, 2710–2723. 10.1002/mrm.29161 35049104 PMC9306807

[IMAG.a.143-b89] Mills, R. (1973). Self-diffusion in normal and heavy water in the range 1–45°. J Phys Chem, 77, 685–688. 10.1021/j100624a025

[IMAG.a.143-b90] Mori, S., & van Zijl, P. C. M. (1995). Diffusion weighting by the trace of the diffusion tensor within a single scan. Magn Reson Med, 33, 41–52. 10.1002/mrm.1910330107 7891534

[IMAG.a.143-b91] Morris, K. F., & Johnson Jr, C. S. (1992). Diffusion-ordered two-dimensional nuclear magnetic resonance spectroscopy. J Am Chem Soc, 114, 3139–3141. 10.1021/ja00034a071

[IMAG.a.143-b92] Moseley, M. E., Kucharczyk, J., Asgari, H. S., & Norman, D. (1991). Anisotropy in diffusion-weighted MRI. Magn Reson Med, 19, 321–326. 10.1002/mrm.1910190222 1652674

[IMAG.a.143-b93] Narvaez, O., Svenningsson, L., Yon, M., Sierra, A., & Topgaard, D. (2022). Massively multidimensional diffusion-relaxation correlation MRI. Front Phys, 9, 793966. 10.3389/fphy.2021.793966

[IMAG.a.143-b94] Narvaez, O., Yon, M., Jiang, H., Bernin, D., Forssell-Aronsson, E., Sierra, A., & Topgaard, D. (2024). Nonparametric distributions of tensor-valued Lorentzian diffusion spectra for model-free data inversion in multidimensional diffusion MRI. J Chem Phys, 161, 084201. 10.1063/5.0213252 39171708

[IMAG.a.143-b95] Neely, J., & Connick, R. (1970). Rate of water exchange from hydrated magnesium ion. J Am Chem Soc, 92, 3476–3478. 10.1021/ja00714a048

[IMAG.a.143-b96] Neuman, C. H. (1974). Spin echo of spins diffusing in a bounded medium. J Chem Phys, 60, 4508–4511. 10.1063/1.1680931

[IMAG.a.143-b97] Nielsen, J. S., Dyrby, T. B., & Lundell, H. (2018). Magnetic resonance temporal diffusion tensor spectroscopy of disordered anisotropic tissue. Sci Rep, 8, 2930. 10.1038/s41598-018-19475-y 29440724 PMC5811563

[IMAG.a.143-b98] Nilsson, M., Lätt, J., Nordh, E., Wirestam, R., & Ståhlberg, F. (2009). On the effects of varied diffusion time in vivo: Is the diffusion in white matter restricted? Magn Reson Imaging, 27, 176–187. 10.1016/j.mri.2008.06.003 18657924

[IMAG.a.143-b99] Nilsson, M., Szczepankiewicz, F., Lampinen, B., Ahlgren, A., de Almeida Martins, J. P., Lasič, S., Westin, C.-F., & Topgaard, D. (2018). An open-source framework for analysis of multidimensional diffusion MRI data implemented in MATLAB. Proc Intl Soc Mag Reson Med, 26, 5355.

[IMAG.a.143-b100] Nilsson, M., Szczepankiewicz, F., van Westen, D., & Hansson, O. (2015). Extrapolation-based references improve motion and eddy-current correction of high b-value DWI data: Application in Parkinson’s disease dementia. PLoS One, 10, e0141825. 10.1371/journal.pone.0141825 26528541 PMC4631453

[IMAG.a.143-b101] Novello, L., Henriques, R. N., Ianus, A., Feiweier, T., Shemesh, N., & Jovicich, J. (2022). In vivo correlation tensor MRI reveals microscopic kurtosis in the human brain on a clinical 3T scanner. Neuroimage, 254, 119137. 10.1016/j.neuroimage.2022.119137 35339682

[IMAG.a.143-b102] Novikov, D. S., Fieremans, E., Jensen, J. H., & Helpern, J. A. (2011). Random walks with barriers. Nat Phys, 7, 508–514. 10.1038/nphys1936 21686083 PMC3114643

[IMAG.a.143-b103] Packer, K. J., & Rees, C. (1972). Pulsed NMR studies of restricted diffusion. I. Droplet size distributions in emulsions. J Colloid Interface Sci, 40, 206–218. 10.1016/0021-9797(72)90010-0

[IMAG.a.143-b104] Parsons, E. C., Does, M. D., & Gore, J. C. (2003). Modified oscillating gradient pulses for direct sampling of the diffusion spectrum suitable for imaging sequences. Magn Reson Imaging, 21, 279–285. 10.1016/s0730-725x(03)00155-3 12850719

[IMAG.a.143-b105] Parsons Jr, E. C., Does, M. D., & Gore, J. C. (2006). Temporal diffusion spectroscopy: Theory and implementation in restricted systems using oscillating gradients. Magn Reson Med, 55, 75–84. 10.1002/mrm.20732 16342147

[IMAG.a.143-b106] Persson, E., & Halle, B. (2008). Cell water dynamics on multiple time scales. Proc Natl Acad Sci USA, 105, 6266–6271. 10.1073/pnas.0709585105 18436650 PMC2359779

[IMAG.a.143-b107] Portnoy, S., Flint, J. J., Blackband, S. J., & Stanisz, G. J. (2013). Oscillating and pulsed gradient diffusion magnetic resonance microscopy over an extended b-value range: Implications for the characterization of tissue microstructure. Magn Reson Med, 69, 1131–1145. 10.1002/mrm.24325 22576352 PMC3421060

[IMAG.a.143-b108] Prange, M., & Song, Y. Q. (2009). Quantifying uncertainty in NMR *T*_2_ spectra using Monte Carlo inversion. J Magn Reson, 196, 54–60. 10.1016/j.jmr.2008.10.008 18952474

[IMAG.a.143-b109] Provencher, S. W. (1982). A constrained regularization method for inverting data represented by linear algebraic or integral equations. Computer Phys Comm, 27, 213–227. 10.1016/0010-4655(82)90173-4

[IMAG.a.143-b110] Reymbaut, A., Critchley, J., Durighel, G., Sprenger, T., Sughrue, M., Bryskhe, K., & Topgaard, D. (2021). Towards non-parametric diffusion-*T*_1_ characterization of crossing fibers in the human brain. Magn Reson Med, 85, 2815–2827. 10.1002/mrm.28604 33301195 PMC7898694

[IMAG.a.143-b111] Reymbaut, A., Mezzani, P., de Almeida Martins, J. P., & Topgaard, D. (2020a). Accuracy and precision of statistical descriptors obtained from multidimensional diffusion signal inversion algorithms. NMR Biomed, 33, e4267. 10.1002/nbm.4267 32067322

[IMAG.a.143-b112] Reymbaut, A., Zheng, Y., Li, S., Sun, W., Xu, H., Daimiel Naranjo, I., Thakur, S., Pinker-Domenig, K., Rajan, S., Vanugopal, V. K., Mahajan, V., Mahajan, H., Critchley, J., Durighel, G., Sughrue, M., Bryskhe, K., & Topgaard, D. (2020b). Clinical research with advanced diffusion encoding methods in MRI. In D. Topgaard (Ed.), Advanced diffusion encoding methods in MRI (pp. 406–429). Royal Society of Chemistry. 10.1039/9781788019910-00406

[IMAG.a.143-b113] Reynaud, O., Winters, K. V., Hoang, D. M., Wadghiri, Y. Z., Novikov, D. S., & Kim, S. G. (2016a). Surface-to-volume ratio mapping of tumor microstructure using oscillating gradient diffusion weighted imaging. Magn Reson Med, 76(1), 237–247. 10.1002/mrm.25865 26207354 PMC4724565

[IMAG.a.143-b114] Reynaud, O., Winters, K. V., Hoang, D. M., Wadghiri, Y. Z., Novikov, D. S., & Kim, S. G. (2016b). Pulsed and oscillating gradient MRI for assessment of cell size and extracellular space (POMACE) in mouse gliomas. NMR Biomed, 29, 1350–1363. 10.1002/nbm.3577 27448059 PMC5035213

[IMAG.a.143-b115] Rosenberg, J. T., Grant, S. C., & Topgaard, D. (2022). Nonparametric 5D **D**-*R*_2_ distribution imaging with single-shot EPI at 21.1 T: Initial results for in vivo rat brain. J Magn Reson, 341, 107256. 10.1016/j.jmr.2022.107256 35753184 PMC9339475

[IMAG.a.143-b116] Rumble, J. (Ed.). (2021). CRC handbook of chemistry and physics, 102 ed. CRC Press. https://hero.epa.gov/hero/index.cfm/reference/details/reference_id/4731459

[IMAG.a.143-b117] Saliani, A., Perraud, B., Duval, T., Stikov, N., Rossignol, S., & Cohen-Adad, J. (2017). Axon and myelin morphology in animal and human spinal cord. Front Neuroanat, 11, 129. 10.3389/fnana.2017.00129 29311857 PMC5743665

[IMAG.a.143-b118] Schachter, M., Does, M. D., Anderson, A. W., & Gore, J. C. (2000). Measurements of restricted diffusion using an oscillating gradient spin-echo sequence. J Magn Reson, 147, 232–237. 10.1006/jmre.2000.2203 11097814

[IMAG.a.143-b119] Simoes, M. C., Hughes, K. J., Ingham, D. B., Ma, L., & Pourkashanian, M. (2017). Estimation of the thermochemical radii and ionic volumes of complex ions. Inorg Chem, 56, 7566–7573. 10.1021/acs.inorgchem.7b01205 28613068

[IMAG.a.143-b120] Sjölund, J., Szczepankiewicz, F., Nilsson, M., Topgaard, D., Westin, C.-F., & Knutsson, H. (2015). Constrained optimization of gradient waveforms for generalized diffusion encoding. J Magn Reson, 261, 157–168. 10.1016/j.jmr.2015.10.012 26583528 PMC4752208

[IMAG.a.143-b121] Slator, P. J., Palombo, M., Miller, K. L., Westin, C. F., Laun, F., Kim, D., Haldar, J. P., Benjamini, D., Lemberskiy, G., de Almeida Martins, J. P., & Hutter, J. (2021). Combined diffusion-relaxometry microstructure imaging: Current status and future prospects. Magn Reson Med, 86, 2987–3011. 10.1002/mrm.28963 34411331 PMC8568657

[IMAG.a.143-b122] Song, Y., Ly, I., Fan, Q., Nummenmaa, A., Martinez-Lage, M., Curry, W. T., Dietrich, J., Forst, D. A., Rosen, B. R., Huang, S. Y., & Gerstner, E. R. (2022). Measurement of full diffusion tensor distribution using high-gradient diffusion MRI and applications in diffuse gliomas. Front Phys, 10, 813475. 10.3389/fphy.2022.813475

[IMAG.a.143-b123] Song, Y.-Q., Venkataramanan, L., Kausik, R., & Heaton, N. (2016). Two-dimensional NMR of diffusion and relaxation. In R. Valiullin (Ed.), Diffusion NMR of confined systems: Fluid transport in porous solids and heterogeneous materials (pp. 111–155). Royal Society of Chemistry. 10.1039/9781782623779-00111

[IMAG.a.143-b124] Stejskal, E. O. (1965). Use of spin echoes in a pulsed magnetic-field gradient to study anisotropic, restricted diffusion and flow. J Chem Phys, 43, 3597–3603. 10.1063/1.1696526

[IMAG.a.143-b125] Stejskal, E. O., & Tanner, J. E. (1965). Spin diffusion measurements: Spin echoes in the presence of a time-dependent field gradient. J Chem Phys, 42, 288–292. 10.1063/1.1695690

[IMAG.a.143-b126] Stepišnik, J. (1981). Analysis of NMR self-diffusion measurements by a density matrix calculation. Physica B, 104, 305–364. 10.1016/0378-4363(81)90182-0

[IMAG.a.143-b127] Stepišnik, J. (1993). Time-dependent self-diffusion by NMR spin-echo. Physica B, 183, 343–350. 10.1016/0921-4526(93)90124-O

[IMAG.a.143-b128] Stepišnik, J., & Callaghan, P. T. (2000). The long time tail of molecular velocity correlation in a confined fluid: Observation by modulated gradient spin-echo NMR. Physica B, 292, 296–301. 10.1016/S0921-4526(00)00469-5

[IMAG.a.143-b129] Stepišnik, J., Lasič, S., Mohoric, A., Sersa, I., & Sepe, A. (2006). Spectral characterization of diffusion in porous media by the modulated gradient spin echo with CPMG sequence. J Magn Reson, 182, 195–199. 10.1016/j.jmr.2006.06.023 16844392

[IMAG.a.143-b130] Stilbs, P. (1987). Fourier transform pulsed-gradient spin-echo studies of molecular diffusion. Prog Nucl Magn Reson Spectrosc, 19, 1–45. 10.1016/0079-6565(87)80007-9

[IMAG.a.143-b131] Szczepankiewicz, F., Lasič, S., van Westen, D., Sundgren, P. C., Englund, E., Westin, C.-F., Ståhlberg, F., Lätt, J., Topgaard, D., & Nilsson, M. (2015). Quantification of microscopic diffusion anisotropy disentangles effects of orientation dispersion from microstructure: Applications in healthy volunteers and in brain tumors. Neuroimage, 104, 241–252. 10.1016/j.neuroimage.2014.09.057 25284306 PMC4252798

[IMAG.a.143-b132] Tan, E. T., Shih, R. Y., Mitra, J., Sprenger, T., Hua, Y., Bhushan, C., Bernstein, M. A., McNab, J. A., DeMarco, J. K., Ho, V. B., & Foo, T. K. F. (2020). Oscillating diffusion-encoding with a high gradient-amplitude and high slew-rate head-only gradient for human brain imaging. Magn Reson Med, 84, 950–965. 10.1002/mrm.28180 32011027 PMC7180099

[IMAG.a.143-b133] Tanner, J. E. (1979). Self diffusion of water in frog muscle. Biophys J, 28, 107–116. 10.1016/S0006-3495(79)85162-0 318065 PMC1328613

[IMAG.a.143-b134] Tax, C. M. W. (2020). Estimating chemical and microstructural heterogeneity by correlating relaxation and diffusion. In D. Topgaard (Ed.), Advanced diffusion encoding methods in MRI (pp. 186–227). Royal Society of Chemistry. 10.1039/9781788019910-00186 33591682

[IMAG.a.143-b135] Tetreault, P., Harkins, K. D., Baron, C. A., Stobbe, R., Does, M. D., & Beaulieu, C. (2020). Diffusion time dependency along the human corpus callosum and exploration of age and sex differences as assessed by oscillating gradient spin-echo diffusion tensor imaging. Neuroimage, 210, 116533. 10.1016/j.neuroimage.2020.116533 31935520

[IMAG.a.143-b136] Tofts, P. (2003). Quantitative MRI of the brain: Measuring changes caused by disease. John Wiley. https://onlinelibrary.wiley.com/doi/book/10.1002/0470869526

[IMAG.a.143-b137] Topgaard, D. (2016a). Director orientations in lyotropic liquid crystals: Diffusion MRI mapping of the Saupe order tensor. Phys Chem Chem Phys, 18, 8545–8553. 10.1039/c5cp07251d 26948308

[IMAG.a.143-b138] Topgaard, D. (2016b). NMR methods for studying microscopic diffusion anisotropy. In R. Valiullin (Ed.), Diffusion NMR of confined systems: Fluid transport in porous solids and heterogeneous materials (pp. 226–259). Royal Society of Chemistry. 10.1039/9781782623779-00226

[IMAG.a.143-b139] Topgaard, D. (2017). Multidimensional diffusion MRI. J Magn Reson, 275, 98–113. 10.1016/j.jmr.2016.12.007 28040623

[IMAG.a.143-b140] Topgaard, D. (2019a). Diffusion tensor distribution imaging. NMR Biomed, 32, e4066. 10.1002/nbm.4066 30730586 PMC6593682

[IMAG.a.143-b141] Topgaard, D. (2019b). Multiple dimensions for random walks. J Magn Reson, 306, 150–154. 10.1016/j.jmr.2019.07.024 31307891

[IMAG.a.143-b142] Topgaard, D. (2020). Translational motion of water in biological tissues—A brief primer. In D. Topgaard (Ed.), Advanced diffusion encoding methods in MRI (pp. 1–11). Royal Society of Chemistry. 10.1039/9781788019910-00001

[IMAG.a.143-b143] Topgaard, D. (2025). Validity of the Gaussian phase distribution approximation for analysis of isotropic diffusion encoding applied to restricted diffusion in a cylinder. Magn Reson Lett, 200196. Advance online publication. 10.1016/j.mrl.2025.200196 PMC12640017 41278257

[IMAG.a.143-b144] Topgaard, D., Malmborg, C., & Söderman, O. (2002). Restricted self-diffusion of water in a highly concentrated W/O emulsion studied using modulated gradient spin-echo NMR. J Magn Reson, 156, 195–201. 10.1006/jmre.2002.2556 12165254

[IMAG.a.143-b145] Tournier, J. D., Smith, R., Raffelt, D., Tabbara, R., Dhollander, T., Pietsch, M., Christiaens, D., Jeurissen, B., Yeh, C. H., & Connelly, A. (2019). MRtrix3: A fast, flexible and open software framework for medical image processing and visualisation. Neuroimage, 202, 116137. 10.1016/j.neuroimage.2019.116137 31473352

[IMAG.a.143-b146] Van, A. T., Holdsworth, S. J., & Bammer, R. (2014). In vivo investigation of restricted diffusion in the human brain with optimized oscillating diffusion gradient encoding. Magn Reson Med, 71, 83–94. 10.1002/mrm.24632 23447055 PMC3732532

[IMAG.a.143-b147] Veraart, J., Lemberskiy, G., Baete, S., Novikov, D. S., & Fieremans, E. (2020). Model-based analysis of advanced diffusion data. In D. Topgaard (Ed.), Advanced diffusion encoding methods in MRI (pp. 317–348). Royal Society of Chemistry. 10.1039/9781788019910-00317

[IMAG.a.143-b148] Wadsö, L., Anderberg, A., Åslund, I., & Söderman, O. (2009). An improved method to validate the relative humidity generation in sorption balances. Eur J Pharm Biopharm, 72, 99–104. 10.1016/j.ejpb.2008.10.013 19022384

[IMAG.a.143-b149] Wagshul, M. E., Eide, P. K., & Madsen, J. R. (2011). The pulsating brain: A review of experimental and clinical studies of intracranial pulsatility. Fluids Barriers CNS, 8, 5. 10.1186/2045-8118-8-5 21349153 PMC3042979

[IMAG.a.143-b150] Wennerström, H. (1973). Proton nuclear magnetic resonance lineshapes in lamellar liquid crystals. Chem Phys Lett, 18, 41–44. 10.1016/0009-2614(73)80333-1

[IMAG.a.143-b151] Westin, C.-F., Knutsson, H., Pasternak, O., Szczepankiewicz, F., Özarslan, E., van Westen, D., Mattisson, C., Bogren, M., O’Donnell, L., Kubicki, M., Topgaard, D., & Nilsson, M. (2016). Q-space trajectory imaging for multidimensional diffusion MRI of the human brain. Neuroimage, 135, 345–362. 10.1016/j.neuroimage.2016.02.039 26923372 PMC4916005

[IMAG.a.143-b152] Westin, C.-F., Szczepankiewicz, F., Pasternak, O., Özarslan, E., Topgaard, D., Knutsson, H., & Nilsson, M. (2014). Measurement tensors in diffusion MRI: Generalizing the concept of diffusion encoding. Med Image Comput Comput Assist Interv, 17, 209–216. 10.1007/978-3-319-10443-0_27 25320801 PMC4386881

[IMAG.a.143-b153] Wetscherek, A., Stieltjes, B., & Laun, F. B. (2015). Flow-compensated intravoxel incoherent motion diffusion imaging. Magn Reson Med, 74, 410–419. 10.1002/mrm.25410 25116325

[IMAG.a.143-b154] Whittal, K. P., & MacKay, A. L. (1989). Quantitative interpretation of NMR relaxation data. J Magn Reson, 84, 134–152. 10.1016/0022-2364(89)90011-5

[IMAG.a.143-b155] Woessner, D. E. (1963). N.M.R. spin-echo self-diffusion measurements on fluids undergoing restricted diffusion. J Phys Chem, 67, 1365–1367. 10.1021/j100800a509

[IMAG.a.143-b156] Wu, D., Martin, L. J., Northington, F. J., & Zhang, J. (2019). Oscillating-gradient diffusion magnetic resonance imaging detects acute subcellular structural changes in the mouse forebrain after neonatal hypoxia-ischemia. J Cereb Blood Flow Metab, 39(7), 1336–1348. 10.1177/0271678X18759859 29436246 PMC6668516

[IMAG.a.143-b157] Xu, J., Li, K., Smith, R. A., Waterton, J. C., Zhao, P., Chen, H., Does, M. D., Manning, H. C., & Gore, J. C. (2012). Characterizing tumor response to chemotherapy at various length scales using temporal diffusion spectroscopy. PLoS One, 7, e41714. 10.1371/journal.pone.0041714 22911846 PMC3404000

[IMAG.a.143-b158] Yon, M., de Almeida Martins, J. P., Bao, Q., Budde, M. D., Frydman, L., & Topgaard, D. (2020). Diffusion tensor distribution imaging of an in vivo mouse brain at ultra-high magnetic field by spatiotemporal encoding. NMR Biomed, 33, e4355. 10.1002/nbm.4355 32812669 PMC7583469

[IMAG.a.143-b159] Yon, M., Narvaez, O., Topgaard, D., & Sierra, A. (2024). In vivo rat brain mapping of multiple gray matter water populations using nonparametric **D**(*ω*)-*R*_1_-*R*_2_ distributions MRI. NMR Biomed, 38, e5286. 10.1002/nbm.5286 39582188 PMC11628177

[IMAG.a.143-b160] Zhang, H., Schneider, T., Wheeler-Kingshott, C. A., & Alexander, D. C. (2012). NODDI: Practical in vivo neurite orientation dispersion and density imaging of the human brain. Neuroimage, 61, 1000–1016. 10.1016/j.neuroimage.2012.03.072 22484410

[IMAG.a.143-b161] Zimmerman, J. R. (1954). Proton relaxation in Mn++ aqueous solutions. J Chem Phys, 22, 950–950. 10.1063/1.1740232

